# An improved African vultures optimization algorithm based on tent chaotic mapping and time-varying mechanism

**DOI:** 10.1371/journal.pone.0260725

**Published:** 2021-11-30

**Authors:** Jiahao Fan, Ying Li, Tan Wang

**Affiliations:** 1 College of Computer Science and Technology, Jilin University, Changchun, China; 2 Key Laboratory of Symbolic Computation and Knowledge Engineering of Ministry of Education, Jilin University, Changchun, China; 3 Northeast Asian Research Center, Jilin University, Changchun, China; Torrens University Australia, AUSTRALIA

## Abstract

Metaheuristic optimization algorithms are one of the most effective methods for solving complex engineering problems. However, the performance of a metaheuristic algorithm is related to its exploration ability and exploitation ability. Therefore, to further improve the African vultures optimization algorithm (AVOA), a new metaheuristic algorithm, an improved African vultures optimization algorithm based on tent chaotic mapping and time-varying mechanism (TAVOA), is proposed. First, a tent chaotic map is introduced for population initialization. Second, the individual’s historical optimal position is recorded and applied to individual location updating. Third, a time-varying mechanism is designed to balance the exploration ability and exploitation ability. To verify the effectiveness and efficiency of TAVOA, TAVOA is tested on 23 basic benchmark functions, 28 CEC 2013 benchmark functions and 3 common real-world engineering design problems, and compared with AVOA and 5 other state-of-the-art metaheuristic optimization algorithms. According to the results of the Wilcoxon rank-sum test with 5%, among the 23 basic benchmark functions, the performance of TAVOA has significantly better than that of AVOA on 13 functions. Among the 28 CEC 2013 benchmark functions, the performance of TAVOA on 9 functions is significantly better than AVOA, and on 17 functions is similar to AVOA. Besides, compared with the six metaheuristic optimization algorithms, TAVOA also shows good performance in real-world engineering design problems.

## 1. Introduction

In the industrial field, optimization problems are often encountered. An optimization problem gives a set of parameters to make the design goal reach the optimal value under certain constraints. Therefore, the optimization problem is an NP-hard problem as it balances time and accuracy [1]. In recent years, due to the continuous development of computer hardware, an increasing number of industrial fields have no longer been limited by computing performance but have begun to pursue higher quality-solutions. Therefore, approximation algorithms have been increasingly applied to solve various real-world complex optimization problems, such as path planning [2], path pursuit [3], feature selection [4], and power dispatching [5], and have achieved good results [6].

The existing approximate algorithm methods are divided into heuristic optimization algorithms and metaheuristic optimization algorithms [7]. The heuristic algorithm needs to give a feasible solution to the problem to be optimized under the specified time complexity and space complexity. However, heuristic algorithms are constructed based on experience, and thus they can only solve specific optimization problems [8]. The result is that a heuristic optimization algorithm can easily fall into a locally optimal solution and has received less attention [9]. Therefore, the deviation between the feasible solution and the optimal solution obtained by the heuristic optimization algorithm is often unpredictable. The metaheuristic optimization algorithm is an improvement of the heuristic optimization algorithm, and it combines a random strategy and a local search strategy. Therefore, although a metaheuristic optimization algorithm cannot guarantee that the feasible solution obtained is the globally optimal solution, it can address various challenging complex optimization problems without worrying about falling into a locally optimal solution [10]. Therefore, an increasing number of scholars have begun to pay attention to and deeply study metaheuristic optimization algorithms.

According to different design inspirations, metaheuristic optimization algorithms are divided into single solution-based metaheuristic optimization algorithms and population-based metaheuristic optimization algorithms [11]. Since only one solution of the single solution-based metaheuristic optimization algorithm participates in the optimization process, the search for the whole solution space is not thorough enough, which result in the algorithm easily falling into a locally optimal solution [12]. The population-based metaheuristic algorithm involves a population in the optimization process. Individuals in the population can not only explore more solution space but also exchange solution information with one another, and thus it is easier to eliminate local trapping [13]. In particular, a nature-inspired metaheuristic optimization algorithm can better balance the exploration and exploitation stage in the optimization process [14]. Therefore, a nature-inspired metaheuristic optimization algorithm can use the exploration ability to avoid falling into a locally optimal solution and use the exploitation ability to make each solution converge toward a better goal [15]. Therefore, nature-inspired metaheuristic optimization algorithms have been widely proposed in recent years.

According to different inspired behaviors, nature-inspired metaheuristic optimization algorithms can be divided into four categories: evolution-based, swarm intelligence-based, physics-based and human behavior-related [11]. In 1983, Kirkpatrick et al. introduced the idea of annealing into optimization problems and proposed a simulated annealing algorithm (SA) [16]. SA finds a feasible solution by dynamically adjusting the temperature according to the value of the fitness function. However, SA depends too much on the initial value, and as a result it will converge too slowly or fall into a locally optimal solution [17]. In 1992, inspired by natural selection in Darwin’s theory of biological evolution, Holland proposed a genetic algorithm (GA) [18]. GAs encode a feasible solution into a gene fragment representation and then update the genes through selection, crossover, mutation and other operations. However, the GA algorithm has a coding and decoding process. In this process, the selection of feasible solutions is limited, which limits the local search ability, resulting in low accuracy in solving continuous optimization problems [19]. Particle swarm optimization (PSO) is the earliest metaheuristic algorithm based on swarm intelligence. It was proposed by Kennedy and Eberhart and inspired by bird foraging behavior in 1995 [20]. The PSO algorithm is still widely used in various fields because its effect is good and its principle is easy to understand. However, for the more complex optimization problems, the solution found by a PSO cannot meet the current high-precision requirements [21]. At present, the popular-human behavior related metaheuristic algorithm is the teaching–learning-based optimization algorithm (TLBO), which was proposed by Rao et al. in 2012 [22]. TLBO is a global search through learners’ learning from teachers and mutual learning between learners. However, TLBO easily falls into local optimization, resulting in premature convergence of the algorithm [23].

However, since 2009, most researchers, such as Yang and Mirjalili have begun to focus on swarm intelligence-based metaheuristic algorithms, which are easier to understand. In addition, the swarm intelligence-based metaheuristic algorithm has a better effect and faster convergence than other metaheuristic algorithms. In 2009, cuckoo search (CS) was proposed through cuckoo breeding behavior and flight behavior [24]. In the same year, the firefly algorithm (FA) was proposed by simulating the behavior of fireflies in attracting other fireflies [25]. In 2010, the bat algorithm (BA) was proposed by modeling bat foraging behavior [26]. In 2014, the flower pollination algorithm (FPA) was proposed by simulating the self-pollination behavior and cross-pollination behavior of flowers [27]. In 2014, the grey wolf optimizer (GWO) was proposed according to the population living habits and predation behavior of wolves [28]. In 2015, Mirjalili proposed the ant lion optimizer (ALO) by simulating the predation behavior of ant lions [29]. In the same year, Mirjalili also proposed a moth-flame optimizer (MFO) through moth behavior [30]. In 2016, Mirjalili also proposed the sine cosine algorithm (SCA) [31], dragonfly algorithm (DA) [32] and whale optimization algorithm (WOA) [33]. As the latest new nature-inspired metaheuristic algorithm proposed by Mirjalili and his collaborators in August 2021, the African vultures optimization algorithm (AVOA) has great research value [7].

Although researchers around the world have proposed a variety of metaheuristic optimization algorithms according to biological habits or natural theory, the exploration ability and exploitation ability of these metaheuristic optimization algorithms are still difficult to balance. Therefore, researchers propose different improvement methods based on the existing metaheuristic optimization algorithms according to the problems to be solved.

Cuong-Le et al. proposed an algorithm called new movement strategy cuckoo search (NMS-CS) based on CS to improve the performance of CS for solving optimization problems [34]. To improve the accuracy of the NMS-CS algorithm and avoid the NMS-CS algorithm falling into local optimization, a new movement strategy is proposed to modify the step size of cuckoo in position update. In order to further improve the performance of FA in global optimization problems and obtain better results, Nand et al. proposed an improved firefly algorithm called FA-CMAES [35]. In FA-CMAES, a new step parameter is proposed to improve the exploitation ability of the algorithm, and the covariance matrix adaptation evolution strategy is embedded to improve the diversity of the population. In order to solve the problem of controller tuning, Li et al. improved the original bat algorithm and named the improved algorithm as CMOBA, which was extended to the multi-objective field [36]. In CMOBA, in order to speed up the convergence of the algorithm, a candidate evolution strategy is proposed, and in order to effectively balance the convergence and diversity of the algorithm, a pairwise competition mechanism is designed. In order to solve unconstrained minimization problems and real-world engineering problems, Ozsoydan and Baykasoglu proposed chaos and intensification enhanced flower pollution algorithm [37]. In this algorithm, three chaotic maps are applied to the population initialization of FPA. In addition, a new step function is designed in the algorithm to enhance the local search ability and global search ability of FPA. Tang HW et al. applied an improved GWO which is named RGWO to the multi-robot cooperative target search problem in the unknown environment [38]. In RGWO, the best learning strategy and adaptive inertial weight method are applied to enhance the exploitation ability of the algorithm and maintain the diversity of the population respectively. In addition, in order to prevent RGWO from falling into local traps, adaptive speed adjustment strategy and escape mechanism are used. In order to solve the problem that the original ALO is easy to fall into the local optimal solution, Dong et al. used the dynamic opposite learning strategy and the dynamic random walk method based on dynamic random number to improve the ALO [39]. Because of the lack of population diversity and global search ability of the original MFO, Li et al. applied the flame generation mechanism based on opposition-based learning and differential evolution algorithm and the local search mechanism based on shuffled frog leaping algorithm to the original MFO and proposed an improved MFO called ODSFMFO [40]. In order to enhance the performance of SCA in large-scale global optimization problems, Li et al. proposed a dynamic sine cosine algorithm (DSCA) by designing nonlinear curves [41]. Tian et al. proposed an adventure circuitous strategy, in which an individual will change the flight direction when it falls into a local optimal solution, and apply it to DA [42]. In order to enhance the search performance of WOA in solving high-dimensional problems and improve its efficiency, Zhang and Wen used random opposition learning in the initialization process of WOA to increase the diversity of the population, so as to improve the global search ability of the algorithm, and designed two strategies of random differential disturbance and switching parameter tuning to improve the local search ability of the algorithm [43].

Compared with other metaheuristic algorithms, AVOA has a more comprehensive exploration mechanism and exploitation mechanism. The use of a random strategy increases the exploration ability of the exploitation mechanism and increases the exploitation ability of the exploration mechanism. This approach can not only ensure that AVOA does not fall into local optima and has fast convergence but also ensure that AVOA is not too divergent.

However, even though AVOA has considered the balance between exploration ability and exploitation ability in its design, there are still three shortcomings. First, although exploitation has added a certain exploitation mechanism to accelerate the convergence speed in the early exploration process, it will affect the individual’s global search in the solution space. Without a more comprehensive global search, AVOA will fall into a locally optimal solution in a later stage. Second, AVOA uses only the best two individual-pieces of information in the population in the exploration stage but does not use the individual’s own information. This approach leads to the slow convergence speed of AVOA in the early stage. Therefore, when solving some problems with low time consumption requirements or high real-time requirements, finding a feasible solution cannot meet the requirements. Third, in the later exploitation stage of AVOA, it is considered that the first good solution and the second good solution have the same impact on other individuals. However, this assumption cannot balance the exploration ability and considered ability of AVOA, which leads to the lack of exploration ability in the early stage and the lack of exploitation ability in the later stage.

Therefore, to solve the above three shortcomings of AVOA, this paper proposes an improved African vulture optimization algorithm based on tent chaotic mapping and time-varying mechanism (TAVOA). First, to make TAVOA have a more comprehensive global exploration ability in the early stage, tent chaos is applied to the initialization of the population of TAVOA. In this way, each individual can be more evenly distributed in each position in the solution space during initialization to improve the exploration ability of TAVOA. Second, the individual’s locally optimal solution is recorded in TAVOA for individual location updating in the exploration stage. In this way, individual historical information can be used to enhance the local exploitation ability of TAVOA, and a good feasible solution can be obtained in a short time. In addition, two time-varying coefficients that vary with the number of iterations are designed. One of the coefficients decreases with the number of iterations, which is used to measure the impact of the best individual on the current individual. Another coefficient increases with the number of iterations, which is used to measure the impact of individual historical optimization on the current individual. This approach can balance the exploration ability and exploitation ability of the algorithm. Third, two other time-varying coefficients that vary with the number of iterations are also designed in the exploitation stage of TAVOA. Similarly, one of the coefficients decreases with the number of iterations, which is used to measure the impact of the best individual on the current individual. Another coefficient increases with the number of iterations, which is used to measure the impact of the second-best individual on the current individual. In this way, we can ensure sufficient exploration ability in the early stage and sufficient exploitation ability in the later stage, in such a way that TAVOA can obtain better results.

The rest of this paper is organized as follows. In the Section 2, the design principle and details of the original AVOA are introduced. In the Section 3, three improvements in TAVOA are described, and the pseudo code and flow chart of TAVOA are given. In Section 4, in order to verify the efficiency and effectiveness of TAVOA, TAVOA is tested in 23 common benchmark functions and 28 CEC 2013 benchmark functions. The experimental results are compared not only with AVOA, but also with other five state-of-the-art metaheuristic optimization algorithms. Finally, the shortcomings of TAVOA and the future work of this paper are introduced in Section 5.

## 2. AVOA

AVOA is a new nature-inspired metaheuristic algorithm proposed by Abdollahzadeh et al. in 2021, and it has been applied in many practical engineering projects [7]. AVOA was proposed by simulating and modeling the foraging behavior and living habits of African vultures. In AVOA, the living habits and foraging behavior of African vultures are simulated using the following criteria.

1. There are *N* vultures in the African vultures population, and the size of *N* is set by the algorithm user according to the actual situation. The position space of each vulture is *D* dimension, and the size of *D* depends on the dimension of the problem applied. Similarly, according to the complexity of the problem to be solved, it is necessary to set a maximum number of iterations *T* in advance, which indicate the maximum number of actions of the vulture. Therefore, the position of each vulture *i*(1≤*i*≤*N*) at different iterations *t*(1≤*t*≤*T*) can be expressed as Eq (1).
$$X_{i}^{t}=[x_{i1}^{t},\cdots ,x_{id}^{t},\cdots ,x_{iD}^{t}]$$ (1)
2. According to the living habits of African vultures, the vultures in the population are divided into three groups. If the fitness value of the feasible solution is used to measure the quality position of the vultures, the first group is to find the best feasible solution among all vultures. The second group is that the feasible solution is the second best among all vultures. In addition to the above two vulture groups, the remaining vultures are divided into the third group.
3. The vulture’s foraging habit is through the population together. Therefore, different types of vultures play different roles in the population.
4. Similarly, if it is assumed that the fitness value of the feasible solution in the population can represent the advantages and disadvantages of vultures, the weakest and hungriest vultures correspond to the worst vultures at present. In contrast, the strongest and most abundant vulture corresponds to the best vulture at present. In AOVA, all vultures try to get close to the best vultures and stay away from the worst vultures.

Based on the above four codes of conduct, when solving problems, AOVA can be divided into five stages to simulate various vulture behaviors in the foraging stage.

a. Phase 1: Population Grouping

According to the second rule, after initialization or before starting the next action, the vultures need to be grouped according to their quality. The vulture, that corresponds to the best solution is placed in the first group, and the vulture that corresponds to the second best solution is placed in the second group. The remaining vultures are placed in the third group. Since both the first- and second-best vultures have guiding effects, Eq (2) is designed to select which vulture should be moved toward in the current iteration.

$$R_{i}^{t}=\left\{ \begin{array}{c} {BestVulture}_{1}^{t},\;p_{i}^{t}=L_{1}\\ {BestVulture}_{2}^{t},\;p_{i}^{t}=L_{2} \end{array}\right.$$ (2)

where ${BestVulture}_{1}^{t}=[b_{11}^{t},\cdots ,b_{1d}^{t},\cdots ,b_{1D}^{t}]$ means the best vulture, ${BestVulture}_{2}^{t}=[b_{21}^{t},\cdots ,b_{2d}^{t},\cdots ,b_{2D}^{t}]$ means the second best vulture, *L*_1_ and *L*_2_ are two random numbers in the range [0,1], the sum of the two numbers is 1, $p_{i}^{t}$ is obtained according to the roulette wheel strategy, and its calculation formula is shown in Eq (3).

$$p_{i}^{t}=\frac{f_{i}^{t}}{\sum_{i=1}^{m}f_{i}^{t}}$$ (3)

where $f_{i}^{t}$ represents the fitness value of the first group and second group vultures, and *m* represents the total number of first group and second group vultures.

**Fig 1:**
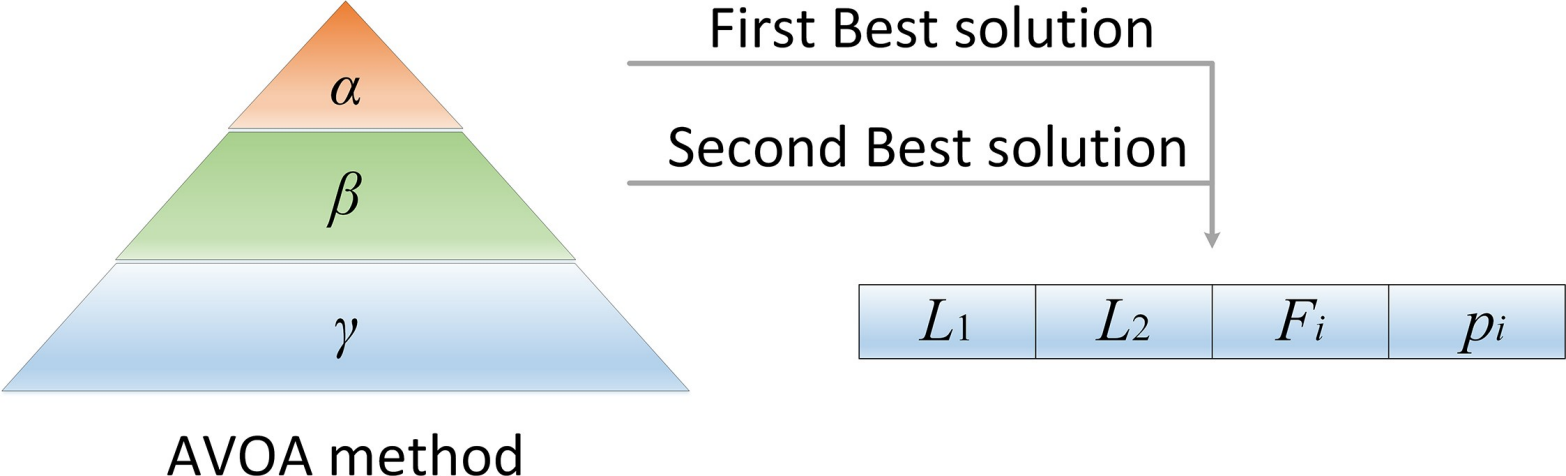
The relationship among the AVOA.

In summary, the relationships between vultures are shown in Fig 1.

where *α* represents the first group of vultures, *β* indicates the second group of vultures, and *γ* indicates the third group of vultures. Then, the target vulture is obtained through relevant parameters.

b. Phase 2: The Hunger of Vultures

If the vulture is not very hungry, it has enough strength to go farther to find food. In contrast, if the vulture feels particularly hungry at present, it does not have enough physical strength to support its long-distance flight. Therefore, hungry vultures will become particularly aggressive, and as a result, they will stay close to the vultures with food instead of looking for food by themselves. Therefore, based on the above behavior, the exploration stage and exploitation stage of vultures can be constructed. The degree of hunger is used as a sign of the transition of vultures from the exploration stage to the exploitation stage. The hunger degree $F_{i}^{t}$ of the *i*th vulture at the *t*th iteration can be calculated by Eq (4).

$$F_{i}^{t}=(2\times {rand}_{i1}^{t}+1)\times z^{t}\times \left(1-\frac{t}{T}\right)+g^{t}$$ (4)

where ${rand}_{i}^{t}$ is a random number in the range of [0,1], *z*^*t*^ is a random number in the range of [−1,1], and *g*^*t*^ is calculated by Eq (5).

$$g^{t}=h^{t}\times ({sin}^{k}(\frac{\pi }{2}\times \frac{t}{T})+\cos(\frac{\pi }{2}\times \frac{t}{T})-1)$$ (5)

where *h*^*t*^ is a random number in the range of [−2,2], and *k* is a parameter set in advance, which indicates the probability of the vulture executing the exploitation stage. A larger *k* indicates that the final optimization stage is more likely to enter the exploration stage. In contrast, a smaller *k* indicates that the final optimization stage is more likely to enter the exploitation stage.

According to the design principle of the formula, $F_{i}^{t}$ will gradually decrease with the increase in the number of iterations, and the decreasing range will continue to increase. Therefore, when $\left|F_{i}^{t}\right|$ is greater than 1, vultures carry out the exploration stage and look for new food in different areas. When $\left|F_{i}^{t}\right|$ is less than 1, vultures go to the exploitation stage to find better food near the current area.

c. Phase 3: Exploration Stage

In nature, vultures have very good eyesight, and thus, they can efficiently find food and dying animals. Therefore, when looking for food, vultures first use a period of time to judge their surrounding environment and then go through a long flight to find the food [44]. In AVOA, the author designs two exploration behaviors and uses a parameter *p*_1_ to decide what type of behavior the vulture will take this time. This parameter *p*_1_ is given with the initialization of the algorithm, and the range is [0,1].

AVOA determines which exploration method the vulture adopts according to a random number, which is in the range [0,1] and is greater than or less than *p*_1_. The exploration stage of the vulture can be expressed as in Eq (6).

$$X_{i}^{t+1}=\left\{ \begin{array}{c} R_{i}^{t}-D_{i}^{t}\times F_{i}^{t},\;p_{1}\geq {rand}_{p1}^{t}\\ R_{i}^{t}-F_{i}^{t}+{rand}_{i2}^{t}\times ((ub-lb)\times {rand}_{i3}^{t}+lb),\;p_{1}<{rand}_{p1}^{t} \end{array}\right.$$ (6)

where $X_{i}^{t+1}$ represents the position of the *i*th vulture at the *t*+1th iteration, ${rand}_{p1}^{t}$, ${rand}_{i2}^{t}$ and ${rand}_{i3}^{t}$ are random numbers that are uniformly distributed in the range [0,1], $R_{i}^{t}$ is obtained according to Eq (2), *F*^*t*^ is calculated according to Eq (4), *ub* and *lb* represent the upper and lower bounds of the solution of the problem, and $D_{i}^{t}$ is calculated by Eq (7) to represent the distance between the vulture and the current optimal vulture.

$$D_{i}^{t}=|C\times R_{i}^{t}-X_{i}^{t}|$$ (7)

where $X_{i}^{t}$ represents the position of the *i*th vulture at the *t*th iteration, and *C* is a random number that is evenly distributed in the range [0,2].

d. Phase 4: Exploitation Stage (Medium)

To avoid the imbalance between the exploration ability and exploitation ability caused by too fast of a transition of the algorithm in the medium term, when the value of $\left|F_{i}^{t}\right|$ is between 0.5 and 1, the vulture will enter the medium-term exploitation stage. In the medium-term exploitation stage, a parameter *p*_2_ with a range of [0,1] is still used. This parameter is used to determine whether the vulture performs food competition or rotating flight. Therefore, when entering the medium-term exploitation stage, a random number ${rand}_{p2}^{t}$ in the range [0,1] will be randomly generated before the vultures act. When ${rand}_{p2}^{t}$ is greater than or equal to parameter *p*_2_, the vultures perform food competition. In contrast, when ${rand}_{p2}^{t}$ is less than parameter *p*_2_, the rotating flight behavior is performed.

(1) Food Competition

When the value of $\left|F_{i}^{t}\right|$ is between 0.5 and 1, the result is that the vulture is full and energetic. Therefore, when the vultures gather together at this time, the strong vultures are unwilling to share their food, while the weak vultures try to gather together and attack the strong vultures to obtain food. Based on this behavior, the vultures’ position update formula can be expressed as in Eq (8).

$$X_{i}^{t+1}=D_{i}^{t}\times (F_{i}^{t}+{rand}_{i4}^{t})-d_{i}^{t}$$ (8)

where $D_{i}^{t}$ is calculated by Eq (7), *F*^*t*^ is calculated by Eq (4), ${rand}_{i4}^{t}$ is a random number that is uniformly distributed in the range [0,1], and $D_{i}^{t}$ is calculated by Eq (9).


$$d_{i}^{t}=R_{i}^{t}-X_{i}^{t}$$ (9)

(2) Rotating Flight

When the vulture is full and energetic, the vulture will not only show food competition behavior but also hover at high altitude. AVOA uses a spiral model to model this behavior. Therefore, in the rotating flight behavior, the position update formula of the vultures can be expressed as in Eq (10).

$$X_{i}^{t+1}=R_{i}^{t}-(S_{i1}^{t}+S_{i2}^{t})$$ (10)

where $S_{i1}^{t}$ and $S_{i2}^{t}$ are calculated by Eq (11) and Eq (12), respectively.

$$S_{i1}^{t}=R_{i}^{t}\times \left(\frac{{rand}_{5}^{t}\times X_{i}^{t}}{2\pi }\right)\times \cos\left(X_{i}^{t}\right)$$ (11)

$$S_{i2}^{t}=R_{i}^{t}\times \left(\frac{{rand}_{6}^{t}\times X_{i}^{t}}{2\pi }\right)\times \sin\left(X_{i}^{t}\right)$$ (12)

where ${rand}_{5}^{t}$ and ${rand}_{6}^{t}$ are random numbers uniformly distributed in the range [0,1].

e. Phase 5: Exploitation stage (later)

When the value of $\left|F_{i}^{t}\right|$ is less than 0.5, almost all vultures in the population have been full, but the best two types of vultures have become hungry and weak after long-term exercise. At this time, vultures will attack food, and many types of vultures will gather in the same food source. Therefore, in the later exploitation stage, there is also a parameter *p*_3_ within the range [0,1]. This parameter is used to determine whether vultures perform attack behavior or aggregation behavior. Therefore, when entering the later exploitation stage, a random number ${rand}_{p3}^{t}$ in the range [0,1] will be randomly generated before the vultures act. When ${rand}_{p3}^{t}$ is greater than or equal to parameter *p*_3_, the vultures exhibit aggregation behavior. In contrast, when ${rand}_{p3}^{t}$ is less than parameter *p*_3_, the vulture conducts attack behavior.

(1) Aggregation Behavior

When AVOA is in the late stage, a large number of foods have been digested by vultures. A large number of vultures will gather where there is food, and competition behavior will occur. At this stage, the vultures’ position update formula can be expressed as in Eq (13).

$$X_{i}^{t+1}=\frac{A_{i1}^{t}+A_{i2}^{t}}{2}$$ (13)

where $A_{i1}^{t}$ and $A_{i2}^{t}$ are calculated by Eq (14) and Eq (15), respectively.


$$A_{i1}^{t}={BestVulture}_{1}^{t}-\frac{{BestVulture}_{1}^{t}\times X_{i}^{t}}{{BestVulture}_{1}^{t}-{\left(X_{i}^{t}\right)}^{2}}\times F_{i}^{t}$$ (14)

$$A_{i2}^{t}={BestVulture}_{2}^{t}-\frac{{BestVulture}_{2}^{t}\times X_{i}^{t}}{{BestVulture}_{2}^{t}-{\left(X_{i}^{t}\right)}^{2}}\times F_{i}^{t}$$ (15)

(2) Attack Behavior

Similarly, when AVOA is in the late stage, the vulture will also move toward the best vulture to try to get the little food left. At this stage, the vultures’ position update formula can be expressed as in Eq (16).

$$X_{i}^{t+1}=R_{i}^{t}-|d_{i}^{t}|\times F_{i}^{t}\times Levy(dim)$$ (16)

where $d_{i}^{t}$ is calculated according to Eq (9), *dim* represents the dimension of the problem solution, *Levy*(∙) represents the Lévy flight [32], and its calculation formula is as shown in Eq (17).

$$Levy(dim)=0.01\times \frac{r_{1}\times \sigma }{{\left|r_{2}\right|}^{\frac{1}{\delta }}}$$ (17)

where *r*_1_ and *r*_2_ are random numbers that are evenly distributed in the range [0,1], *δ* is a constant, which is usually set to 1.5, and the calculation formula of *σ* is shown in Eq (18).

$$\sigma ={\left(\frac{\Gamma (1+\delta )\times \sin\left(\frac{\pi \delta }{2}\right)}{\Gamma (1+\delta )\times \delta \times 2^{\left(\frac{\delta -1}{2}\right)}}\right)}^{\frac{1}{\delta }}$$ (18)

where Γ(*x*) = (*x*−1)!.

## 3. Proposed algorithm

Different from other metaheuristic optimization algorithms, AVOA has a clearer exploration mechanism and exploitation mechanism. However, AVOA still has some disadvantages, such as easily falling into a locally optimal solution and having an imbalance between exploration ability and exploitation ability. To make AVOA more widely used and have a better effect, this paper includes three innovations in the proposed TAVOA. First, a tent chaotic map is used to initialize the population, to realize the diversity of the population and avoid the algorithm falling into a locally optimal solution. Second, making full use of the historical optimal vulture information, the algorithm can obtain a better solution in the early stage, and as a result, it can be applied to more engineering fields. Third, the time-varying mechanism is designed to balance the exploration and exploitation ability of TAVOA, in such a way that the algorithm can obtain a better solution.

### 3.1 Tent chaotic mapping for population initialization

Without exception, similar to other metaheuristic optimization algorithms, AVOA uses randomly generated data in the population initialization. However, this approach is not conducive to population diversity. The diversity of the population can affect the convergence speed and the obtained results of the metaheuristic optimization algorithm and can help the metaheuristic optimization algorithm find the globally optimal solution faster [45]. In addition, because the exploration mechanism and exploitation mechanism of AVOA are especially clear, it is necessary to guide the population to move in a better direction as much as possible in the exploration stage. Otherwise, when the algorithm enters the exploitation stage, it will fall into a locally optimal solution due to the lack of early exploration. Therefore, when there is no prior experience to know where the globally optimal solution is in the solution space, the population needs to cover the whole solution space as much as possible. The population initialization of AVOA is also generated randomly, which cannot cover the whole solution space as much as possible. Therefore, AVOA easily falls into local optimization.

Fortunately, chaotic mapping has the characteristics of randomness and ergodicity. These characteristics can maintain the diversity of the population, make the metaheuristic optimization algorithm escape the local trap and improve the global exploration ability of the metaheuristic optimization algorithm. Kaur and Arora used 10 chaotic maps for tuning parameters in whale optimization algorithm in 2018, and a large number of experiments show that tent chaotic map has the best performance among the 10 chaotic maps commonly used at present [46]. It is worth noting that the chaotic sequence generated by the tent chaotic map is flatter and more uniform than that generated by other chaotic maps [47]. In addition, Arora et al. also applied 10 kinds of chaotic maps to the internal search algorithm in 2020, and experiments show that the tent chaotic map improves the performance of the internal search algorithm the most among these 10 chaotic maps, [48]. Similarly, Zarei and Meybodi also applied 13 chaotic maps to learning automata, and experiments still proved that tent chaotic map performed best among them [49].

Therefore, to solve the above problems, tent chaotic mapping is used to initialize the population in TAVOA to make the population cover the whole solution space as much evenly as possible and improve the performance of the algorithm in the exploration stage. Tent chaotic mapping can be expressed by Eq (19).


$$x^{t+1}=tent(x^{t})=\left\{ \begin{array}{c} \frac{x^{t}}{u},\;0\leq x<u\\ \frac{1-x^{t}}{1-u},\;u\leq x\leq 1 \end{array}\right.$$ (19)

According to the existing research, when *u* = 1/2, the uniformity of tent chaotic mapping is the best [50]. Therefore, to obtain a more evenly distributed sequence, *u* = 1/2 is used in this paper. As a result, Eq (19) can be replaced by Eq (20).


$$x^{t+1}=tent(x^{t})=\left\{ \begin{array}{c} 2x^{t},\;0\leq x<0.5\\ 2(1-x^{t}),\;0.5\leq x\leq 1 \end{array}\right.$$ (20)

Although tent chaotic mapping has made the distribution as uniform as possible, tent chaotic mapping still has some disadvantages. The reason is that the byte length of the computer is limited; as a result, when *x* is a value, after a certain number of iterations, the value of *x* will fall into a fixed value. There are two situations that make tent chaotic mapping fall into a nonrandom cycle. One such situation is when the initial value of *x* belongs to {0.2,0.4,0.6,0.8}. In another such case, after calculation, the value of *x* belongs to {0,0.25,0.5,0.75}.

In conclusion, combined with the limited tent chaotic mapping, the population initialization details of TAVOA are shown in Algorithm 1, where *ε* represents a very small random number, and *ub* and *lb* represent the upper and lower bounds of the solution of the problem, respectively.

**Algorithm 1** Population Initialization.

1: Randomly generate a random number *a*_0_ ranging from [*0*,*1*];

2: **while** *a*_0_∈{0.2,0.4,0.6,0.8} **do**

3:    Regenerate random number *a*_0_, and the range is between [*0*,*1*];

4: **end while**

5: **for** each vulture *i* from 1 to *N* **do**

6:    $a_{i}=tent(a_{i-1})=\left\{ \begin{array}{c} 2a_{i-1},\;0\leq a_{i-1}<0.5\\ 2(1-a_{i-1}),\;0.5\leq a_{i-1}\leq 1 \end{array}\right.;$

7:    **while** *a*_*i*_∈{0,0.25,0.5,0.75} **do**

8:        *a*_*i*_ = *a*_*i*_+*ε*;

9:    **end while**

10:    $X_{i}^{0}=a_{i}\times (ub-lb)+lb;$

11: **end for**

### 3.2 Individual history optimal solution

It can be found from Eq (7) that in the exploration stage, when *p*1 is greater than or equal to the random value ${rand}_{p1}^{t}$, the vulture uses only the information of the current optimal vulture. Although we should explore the unknown area to a great extent in the exploration stage, it will cause the algorithm to not converge in the early stage. Therefore, a long period of iterating is needed to reach the exploitation stage before better results can be obtained. However, this circumstance does not meet many engineering problems with high real-time requirements. In addition, if the algorithm is too divergent in the early stage, it will result in insufficient exploitation time in the later stage, which will cause the algorithm to fail to converge and fall into a locally optimal solution. Therefore, to better apply TAVOA in more engineering fields and obtain better solutions, TAVOA recorded the historical optimal solution of each vulture in the exploration stage and used it in the location updating. This approach can not only limit the divergence of the algorithm in the exploration stage but also use the vulture’s own historical information to ensure that the updated solution will not be too bad.

**Fig 2:**
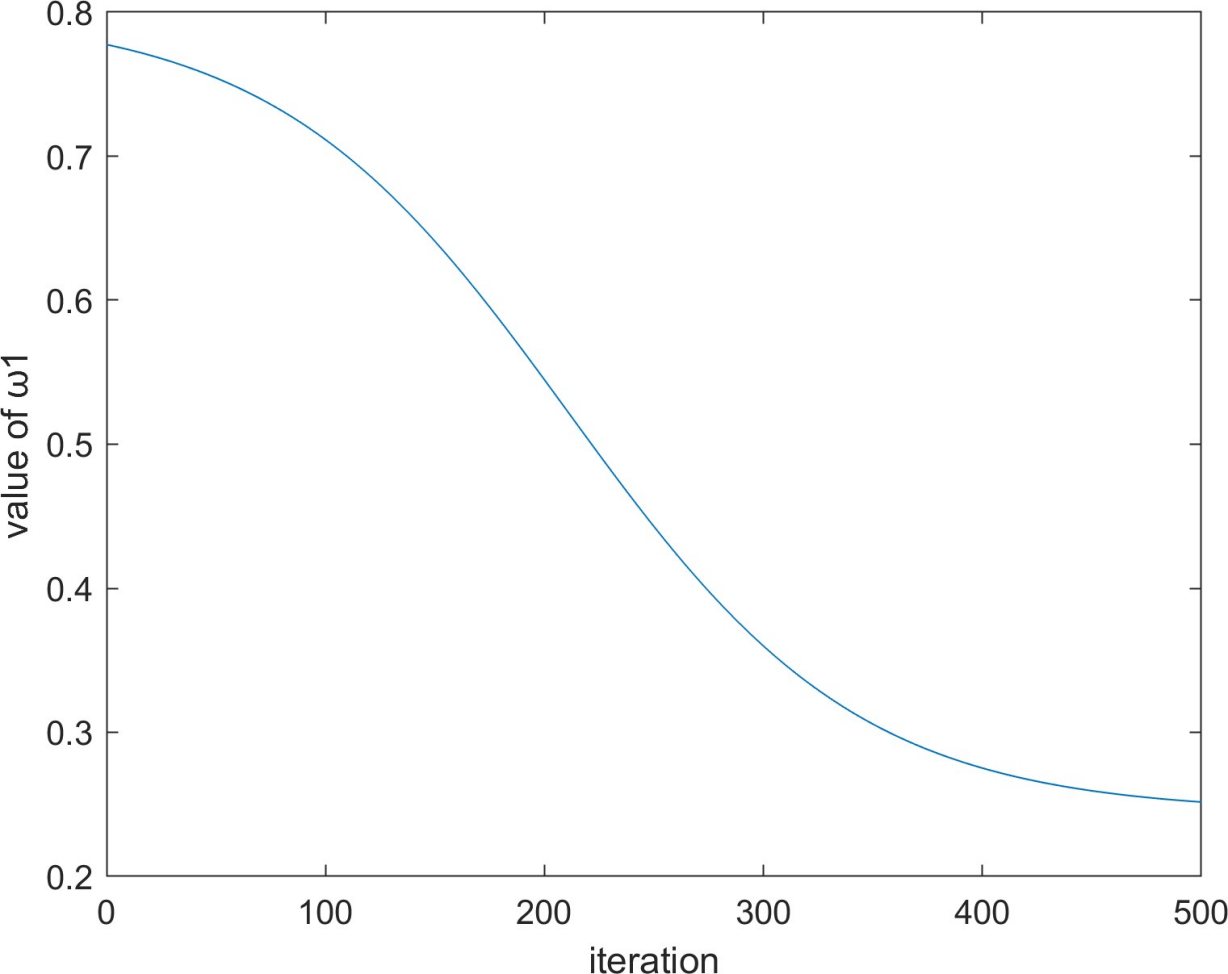
The iteration diagram of $\omega_{1}^{t}$.

**Fig 3:**
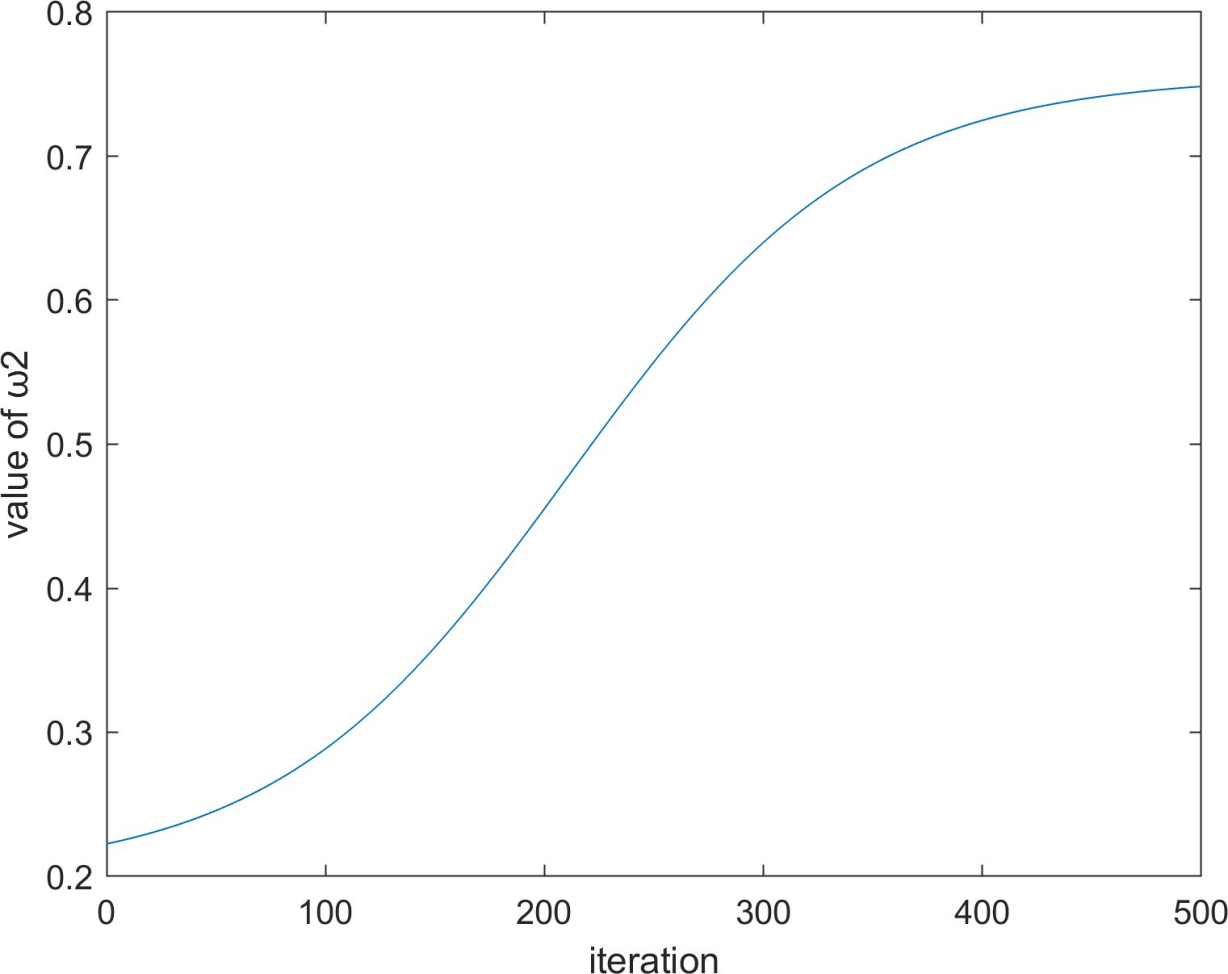
The iteration diagram of $\omega_{2}^{t}$.

Therefore, in the exploration stage, when the random number ${rand}_{p1}^{t}$ is less than or equal to parameter *p*_1_, Eq (7) is replaced by Eq (21).

$$D_{i}^{t}=|\omega_{1}^{t}\times C\times R_{i}^{t}+\omega_{2}^{t}\times C\times P_{i}-X_{i}^{t}|$$ (21)

where *P*_*i*_ is the best position that the *i*th vulture has reached in history, $\omega_{1}^{t}$ and $\omega_{2}^{t}$ are two values that change with the number of iterations, and the calculation formulas of these two values are Eq (22) and Eq (23), respectively.

$$\omega_{1}^{t}=0.2+\frac{1}{1.8+e^{0.015*(\frac{T}{2}-t)}}$$ (22)

$$\omega_{2}^{t}=-\frac{1}{1.8+e^{0.015*(\frac{T}{2}-t)}}-0.8$$ (23)

where *T* represents the maximum number of iterations, and *t* represents the current number of iterations. When *t* = 500, the iteration diagrams of $\omega_{1}^{t}$ and $\omega_{2}^{t}$ with the number of iterations are shown in Figs 2 and 3, respectively.

It is worthwhile to note that in Eq (21), we have added two parameters, $\omega_{1}^{t}$ and $\omega_{2}^{t}$, to control the influence degree of the optimal vulture and the historical optimal vulture respectively. The reason for this design is that the size of |*F*^*t*^| can still be greater than 1 even in the middle and late stages of the algorithm. At this time, the vulture will still enter the exploration stage. However, in the middle and late stages of the algorithm, the optimal vulture cannot affect too many current vultures; otherwise, the algorithm will not converge. Therefore, the time-varying mechanism is used in Eqs (22) and (23). It can be seen from Figs 2 and 3 that the curve is relatively flat in the early and late stages of the algorithm and steep in the middle stage of the algorithm. This outcome occurs because the early and late stages of the algorithm need to be stable, while the middle stage needs a fast transition.

### 3.3 Time-varying mechanism

It can be found from Eq (13) that in the exploitation stage, when the algorithm is in the late stage, AVOA believes that the influence of the first group of vultures and the second group of vultures on the current vulture is the same in the aggregation behavior. However, this is not the case. In the middle of the algorithm, the second group of vultures could be needed to increase the exploration ability and prevent the algorithm from falling into a local optimization. However, in the later stage of the algorithm, if the influence of the first group of vultures and the second group of vultures on the current vultures is the same, the exploration ability and exploitation ability of the algorithm will be similar, resulting in the inability of the algorithm to converge better. Therefore, to enhance the local exploitation ability in the later stage of TAVOA, two factors that control the influence of the first group of vultures and the second group of vultures on the current vulture are introduced. In addition, the time-varying mechanism is applied to these two parameters to further balance the exploration ability and exploitation ability of TAVOA.

**Fig 4:**
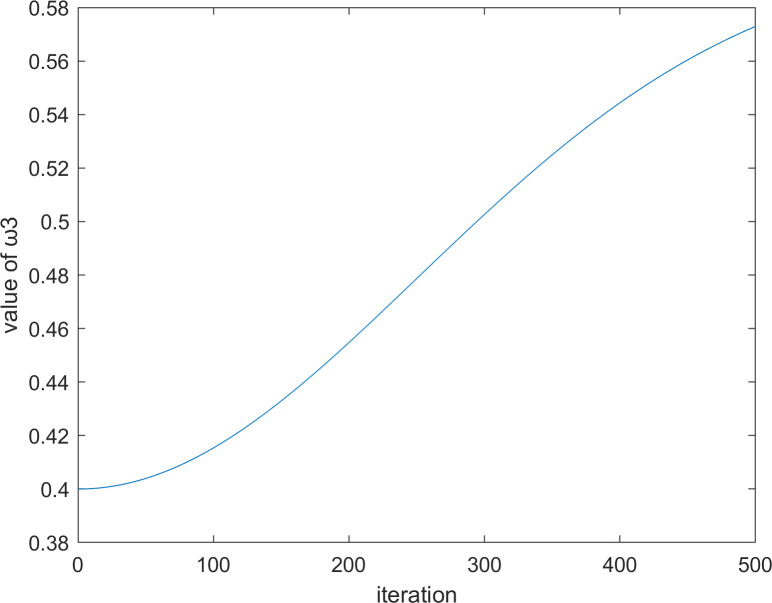
The iteration diagram of $\omega_{3}^{t}$.

**Fig 5:**
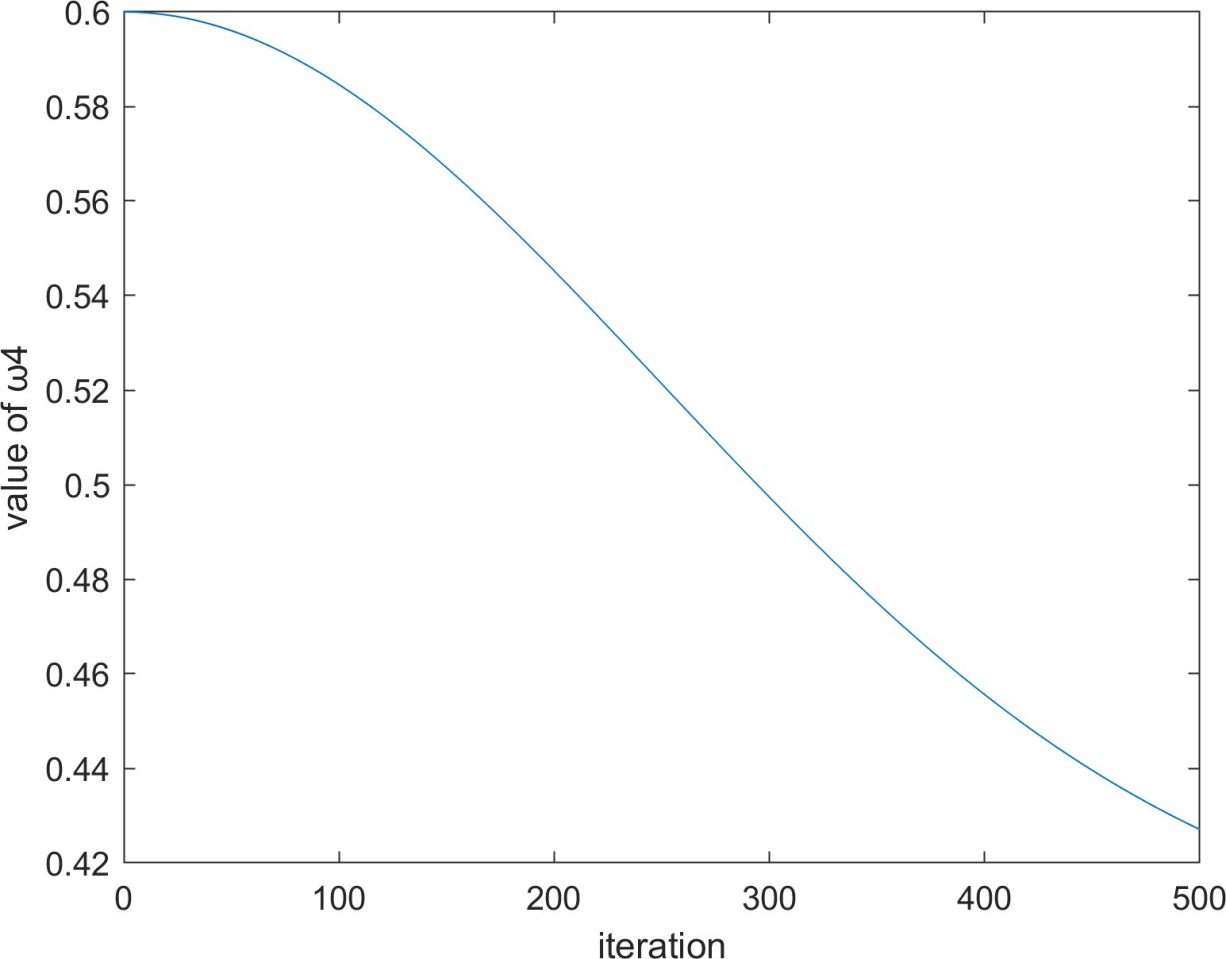
The iteration diagram of $\omega_{4}^{t}$.

Therefore, in the development stage, when the value of $\left|F_{i}^{t}\right|$ is less than 0.5 and ${rand}_{p3}^{t}$ is greater than or equal to parameter *p*_3_, the position update formula of the vulture is changed from the original Eq (13) to Eq (24).

$$X_{i}^{t+1}=\frac{\omega_{3}^{t}{\times A}_{i1}^{t}+\omega_{4}^{t}{\times A}_{i2}^{t}}{2}$$ (24)

where $A_{i1}^{t}$ and $A_{i2}^{t}$ are calculated by Eq (14) and Eq (15), respectively, $\omega_{3}^{t}$ and $\omega_{4}^{t}$ are two values that change with the number of iterations, and the calculation formulas of these two factors are Eq (25) and Eq (26), respectively.

$$\omega_{3}^{t}=-0.2\times e^{-2\times {\left(\frac{t}{T}\right)}^{2}}-0.6$$ (25)

$$\omega_{4}^{t}=0.4+0.2\times e^{-2\times {\left(\frac{t}{T}\right)}^{2}}$$ (26)

where *T* represents the maximum number of iterations, and *t* represents the current number of iterations. When *t* = 500, the iteration diagrams of $\omega_{3}^{t}$ and $\omega_{4}^{t}$ with the number of iterations are shown in Figs 4 and 5, respectively.

It should be noted that if you look at it intuitively, you might feel that Fig 3 is similar to Fig 4, and Fig 2 is similar to Fig 5. However, their differences can be found through comparison. Figs 4 and 5 are not stable as in Figs 2 and 3, but rather are steep in the later stage. This outcome occurs because $\omega_{3}^{t}$ and $\omega_{4}^{t}$ are applied in the later exploitation stage, which needs to quickly convert exploration ability into exploitation ability and maintain sufficient exploitation ability to a certain extent. In addition, in the numerical range, $\omega_{3}^{t}$ and $\omega_{4}^{t}$ are also different from $\omega_{1}^{t}$ and $\omega_{2}^{t}$ because $\omega_{1}^{t}$ and $\omega_{2}^{t}$ are the influence of controlling the optimal vulture and the historical optimal vulture on the current vulture respectively. There could be a large difference between the optimal vulture and the historical optimal vulture, and thus, the values of $\omega_{1}^{t}$ and $\omega_{2}^{t}$ change more. However, $\omega_{3}^{t}$ and $\omega_{4}^{t}$ are the influences of controlling the first group of vultures and the second group of vultures on the current vulture, respectively. In addition, $\omega_{3}^{t}$ and $\omega_{4}^{t}$ are two factors in the later exploitation stage of the algorithm. Therefore, the difference between the first group of vultures and the second group of vultures might not be very large. Therefore, the values of $\omega_{3}^{t}$ and $\omega_{4}^{t}$ change slightly.

### 3.4 Algorithm framework

**Fig 6:**
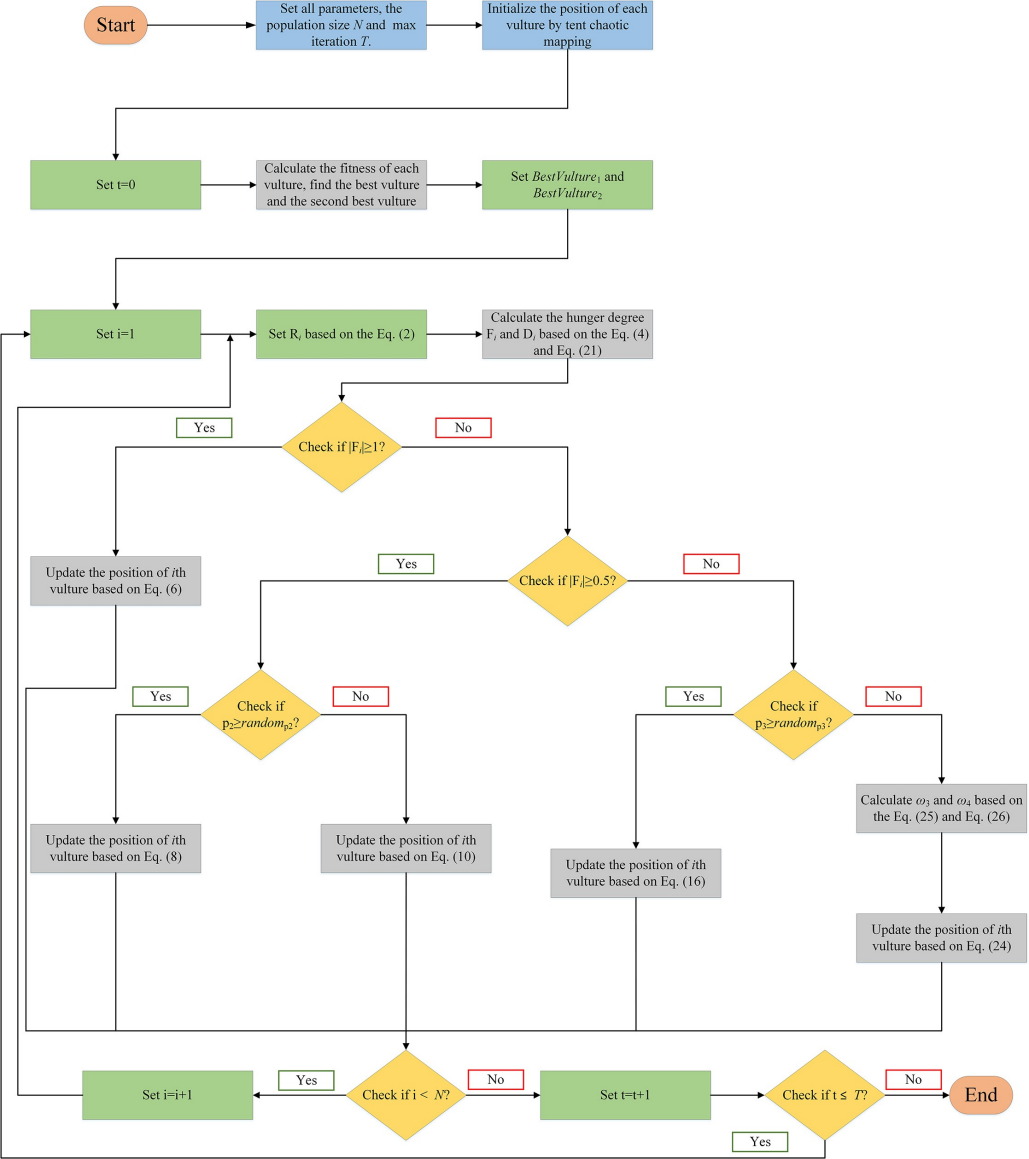
The flowchart of TAVOA.

In summary, the implementation flow of TAVOA proposed in this paper is shown in Fig 6. First, TAVOA does not initialize the population randomly, but is generated according to tent chaotic mapping. Second, the individual history of each vulture will be recorded, and it will have different effects on the vulture at different stages according to the weight. Finally, the time-varying mechanism is applied to the aggregation behavior in the vulture exploitation stage. In the later stage of TAVOA, it enhances its local exploitation ability, accelerates the convergence speed and obtains better results. The pseudo code of TAVOA is shown in Algorithm 2.

**Algorithm 2** Framework of TAVOA.

1:    **Initialize** the population size *N* and maximum number of iterations *T*;

2: **Set** all parameters that need to be given in advance;

2: **Initialize** the position $X_{i}^{0}$ of each vulture according to **Algorithm 1**;

3: **While** (current iteration *t*<*T*) **do**

4:    Calculate the fitness value of each vulture;

5:    Find the best vulture and the second best vulture;

6:    Set the best vulture and the second best vulture to ${BestVulture}_{1}^{t}$ and ${BestVulture}_{2}^{t}$;

7:    **for** each vulture *i* from 1 to *N* **do**

8:        Set $R_{i}^{t}$ based on Eq (2);

9:        Calculate the hunger degree $F_{i}^{t}$ based on Eq (4);

10:        Calculate the $\omega_{1}^{t}$ and $\omega_{2}^{t}$ based on Eqs (22) and (23);

11:        Calculate the distance $D_{i}^{t}$ based on Eq (21);

12:        **if** $|F_{i}^{t}|\geq 1$ **then**

13:            Update the position of *i*th vulture $X_{i}^{t+1}$ based on Eq (6);

14:        **else**

15:            **if** $|F_{i}^{t}|\geq 0.5$ **then**

16:            **if** $p_{2}\geq {rand}_{p2}^{t}$ **then**

17:                Update the position of *i*th vulture $X_{i}^{t+1}$ based on Eq (8);

18:            **else**

19:                Update the position of *i*th vulture $X_{i}^{t+1}$ based on Eq (10);

20:            end if

21:        **else**

22:            **if** $p_{3}\geq {rand}_{p3}^{t}$ **then**

23:                Calculate the $\omega_{3}^{t}$ and $\omega_{4}^{t}$ based on Eqs (25) and (26);

24:                    Update the position of *i*th vulture $X_{i}^{t+1}$ based on Eq (24);

25:                **else**

26:                    Update the position of *i*th vulture $X_{i}^{t+1}$ based on Eq (16);

27:                end if

28:            end if

29:        end if

30:    end for

31: end while

32: **Return** the best vulture

## 4. Experiments and results

To verify the efficiency and effectiveness of TAVOA proposed in this paper, TAVOA is tested on a total of 51 benchmark functions. In this section, in addition to comparison with the original AVOA, five other metaheuristic algorithms are selected for comparison with TAVOA proposed in this paper. The five metaheuristic algorithms are the grasshopper optimization algorithm (GOA) [51], marine predator algorithm (MPA) [52], particle swarm optimization (PSO) [20], slap swarm algorithm (SSA) [53] and whale optimization algorithm (WOA) [33]. PSO is chosen for comparison because it is the most classical and widely used metaheuristic optimization algorithm. The remaining four comparison algorithms were proposed in recent year and have been cited more times.

In addition, the relevant parameters of all comparison algorithms are shown in Table 1. The parameter settings of the comparison algorithms refer to the references written by the algorithms’ proposer. Because TAVOA proposed in this paper is an improved algorithm of AVOA, the parameter settings of TAVOA are consistent with AVOA for fair comparison.

**Table 1:** The parameters setting of all comparison algorithms.

Algorithm	Parameter	Value	Reference
AVOA	*L*_1_	0.8	[7]
	*L*_2_	0.2	
	*k*	2.5	
	*p*_1_	0.6	
	*p*_2_	0.4	
	*p*_3_	0.6	
GOA	*cmax*	1.0	[51]
	*cmin*	0.00004	
	*f*	0.5	
	*l*	1.5	
MPA	*P*	0.5	[52]
	*FADs*	0.2	
PSO	*ω*	[0.2,0.9]	[20]
	*c*_1_	2	
	*c*_2_	2	
SSA	Leader position update probability	0.5	[53]
WOA	Convergence constant $\vec{a}$	[2,0]	[33]
	Spiral factor *b*	1	
TAVOA	*L*_1_	0.8	-
	*L*_2_	0.2	
	*k*	2.5	
	*p*_1_	0.6	
	*p*_2_	0.4	
	*p*_3_	0.6	

All algorithms are implemented in MATLAB 2020b and run in the Windows 10 Home Chinese 64-bit. The CPU is an Intel Core i7-10700 with 2.90 GHz and the RAM is 8.00G.

### 4.1 Benchmark functions and common parameters

In this paper, a total of 51 benchmark functions are applied to test the performance of the metaheuristic optimization algorithm. These 51 benchmark functions can be divided into two sets. One set is 23 basic benchmark functions, which are widely used to test the performance of the metaheuristic optimization algorithms [51]. The details of the first set of 23 basic benchmark functions are shown in Table 2.

**Table 2:** Details of 23 basic benchmark functions.

Type	Function	Dim	Range	*f*_*min*_
**Unimodal benchmark function**	$f1(x)=\sum_{i=1}^{Dim}x_{i}^{2}$	30	[−100,100]^*Dim*^	0
	$f2(x)=\sum_{i=1}^{Dim}\left|x_{i}\right|+\prod_{i=1}^{Dim}\left|x_{i}\right|$	30	[−10,10]^*Dim*^	0
	$f3(x)=\sum_{i=1}^{Dim}{\left(\sum_{j=1}^{i}x_{j}\right)}^{2}$	30	[−100,100]^*Dim*^	0
	$f4(x)={max}_{i}\{|x_{i}|,\;1\leq i\leq Dim\}$	30	[−100,100]^*Dim*^	0
	$f5(x)=\sum_{i=1}^{Dim-1}\left[{100(x_{i+1}-x_{i}^{2})}^{2}+{\left(x_{i}-1\right)}^{2}\right]$	30	[−30,30]^*Dim*^	0
	$f6(x)=\sum_{i=1}^{Dim}{\left(x_{i}+0.5\right)}^{2}$	30	[−100,100]^*Dim*^	0
	$f7(x)=\sum_{i=1}^{Dim}ix_{i}^{4}+random[0,1)$	30	[−1.28,1.28]^*Dim*^	0
**Multi-modal benchmark function**	$f8(x)=\sum_{i=1}^{Dim}-x_{i}sin(\sqrt{\left|x_{i}\right|})$	30	[−500,500]^*Dim*^	−418.9829×*Dim*
	$f9(x)=\sum_{i=1}^{Dim}\left[x_{i}^{2}-10cos(2\pi x_{i})+10\right]$	30	[−5.12,5.12]^*Dim*^	0
	$f10(x)=-20exp\left(-0.2\sqrt{\frac{\sum_{i=1}^{Dim}x_{i}^{2}}{Dim}}\right)-exp\left(\frac{\sum_{i=1}^{Dim}cos(2\pi x_{i})}{Dim}\right)+20+e$	30	[−32,32]^*Dim*^	0
	$f11(x)=\frac{1}{4000}\sum_{i=1}^{Dim}x_{i}^{2}-\prod_{i=1}^{Dim}cos(\frac{x_{i}}{\sqrt{i}})+1$	30	[−600,600]^*Dim*^	0
	$f12(x)=\{10sin(\pi y_{1})+\sum_{i=1}^{Dim-1}{\left(y_{i}-1\right)}^{2}\left[1+10{sin}^{2}(\pi y_{i+1})\right]-{\left(y_{Dim}-1\right)}^{2}\}+\sum_{i=1}^{Dim}u(x_{i},5,100,4)$ $y_{i}=1+(x_{i}+1)/4u(x_{i},a,k,m)=\left\{ \begin{array}{c} k{\left(x_{i}-a\right)}^{m},x_{i}>a\\ 0,-a<x_{i}<a\\ {k(-x_{i}-a)}^{m},x_{i}<-a \end{array}\right.$	30	[−50,50]^*Dim*^	0
	$f13(x)=0.1\{{sin}^{2}(3\pi x_{i})+\sum_{i=1}^{Dim}{\left(x_{i}-1\right)}^{2}\left[1+{sin}^{2}(3\pi x_{i}+1)\right]+{\left(x_{Dim}-1\right)}^{2}\left[1+{sin}^{2}(2\pi x_{Dim})\right]\}+\sum_{i=1}^{Dim}u(x_{i},5,100,4)$	30	[−50,50]^*Dim*^	0
**Fixed-dimension multi-modal benchmark function**	$f14(x)={\left[\frac{1}{500}+\sum_{j=1}^{25}\frac{1}{j+\sum_{i=1}^{2}{\left(x_{i}-a_{ij}\right)}^{6}}\right]}^{-1}$	2	[−65,65]^*Dim*^	1
	$f15(x)=\sum_{i=1}^{11}{\left[a_{i}-\left[x_{1}(b_{i}^{2}+b_{i}x_{2})\right]/\left(b_{i}^{2}+b_{i}x_{3}+x_{4}\right)\right]}^{2}$	4	[−5,5]^*Dim*^	0.00030
	$f16(x)=4x_{1}^{2}-2.1x_{1}^{4}+1/3x_{1}^{6}+x_{1}x_{2}-4x_{2}^{2}+4x_{2}^{4}$	2	[−5,5]^*Dim*^	-1.0316
	$f17(x)={\left(x_{2}-5.1x_{1}^{2}/4\pi^{2}+5x_{1}/\pi -6\right)}^{2}+10(1-1/8\pi )cosx_{1}+10$	2	[−5,5]^*Dim*^	0.398
	$f18(x)=[1+{\left(x_{1}+x_{2}+1\right)}^{2}(19-14x_{1}+3x_{1}^{2}-14x_{2}+6x_{1}x_{2}+3x_{2}^{2})]\times [30+{\left(2x_{1}-3x_{2}\right)}^{2}(18-32x_{1}+12x_{1}^{2}+48x_{2}-36x_{1}x_{2}+27x_{2}^{2})]$	2	[−2,2]^*Dim*^	3
	$f19(x)=-\sum_{i=1}^{4}c_{i}exp(-\sum_{j=1}^{3}{a_{ij}(x_{i}-p_{ij})}^{2})$	3	[1,3]^*Dim*^	-3.86
	$f20(x)=-\sum_{i=1}^{4}c_{i}exp(-\sum_{j=1}^{6}{a_{ij}(x_{i}-p_{ij})}^{2})$	6	[0,1]^*Dim*^	-3.32
	$f21(x)=-\sum_{i=1}^{5}{\left[(X-a_{i}){\left(X-a_{i}\right)}^{T}+c_{i}\right]}^{-1}$	4	[0,10]^*Dim*^	-10.1532
	$f22(x)=-\sum_{i=1}^{7}{\left[(X-a_{i}){\left(X-a_{i}\right)}^{T}+c_{i}\right]}^{-1}$	4	[0,10]^*Dim*^	-10.4028
	$f23(x)=-\sum_{i=1}^{10}{\left[(X-a_{i}){\left(X-a_{i}\right)}^{T}+c_{i}\right]}^{-1}$	4	[0,10]^*Dim*^	-10.5363

However, these 23 basic benchmark functions are relatively simple, and most algorithms can converge in a relatively short number of iterations. Therefore, these 23 test functions are mainly used to test the performance of the algorithm in solving general engineering problems. The other set is the more professional 28 CEC 2013 benchmark functions [54]. These 28 benchmark functions were proposed by Liang JJ et al. in 2013 and have widely and deeply tested the performance of metaheuristic algorithms. In addition, the 28 CEC 2013 benchmark functions can better reflect the ultimate performance of the metaheuristic optimization algorithm.

The first set of basic benchmark functions can be divided into three groups according to the characteristics of functions. The first is unimodal functions (*f*1−*f*7), which is used to test the performance of the algorithm in solving simple problems. The second group is multi-modal functions (*f*8−*f*13), and the third group is multimodal functions with fixed dimension ((*f*14−*f*23). The second and third groups of basic benchmark functions are used to test the diversity of the metaheuristic optimization algorithms.

The second set of CEC 2013 functions can also be divided into three groups according to the characteristics of the functions. The first group is unimodal functions (*F*1−*F*5), corresponding to the functions in this paper (*f*24−*f*28). The second group is multi-modal functions (*F*6−*F*20), corresponding to the functions in this paper (*f*29−*f*43). The third group is the composition functions (*F*21−*F*28), corresponding to the functions in this paper (*f*44−*f*51).

In addition, to make the experiment fairer and more persuasive, the following common parameters are set for all experiments:

1. Number of individuals in the population: *Num* = 30.
2. Dimension of solution space: *Dim* = 30.
3. Number of independent runs: *RunNum* = 30.

The first set of basic benchmark functions more easily converges and achieves stable results. The second set of CEC 2013 benchmark functions is more complex and difficult to converge. Therefore, for the two sets of benchmark functions, the maximum number of iterations in this paper is different.

1. For the first set of 23 basic benchmark functions, the maximum number of iterations: *MaxT*_1_ = 500.
2. For the second set of 28 CEC 2013 benchmark functions, the maximum number of iterations: *MaxT*_2_ = 2×10^5^.

In each function, the best value, the worst value, the mean value and the standard deviation are statistically used for algorithm comparison. In addition, we select the best algorithm according to the mean value of each algorithm based on the results run 30 independently times, and express it in **boldface**. If the mean values are the same, it is considered that the algorithm with a small standard deviation is better. In addition, the Wilcoxon rank-sum test with 5% is used to measure whether there was a significant difference between TAVOA and other comparison algorithms. In the Wilcoxon rank-sum test results, “+/ = /-” is used to indicate that the performance of TAVOA on a function is “better than/similar to/worse than” a comparison algorithm. In order to more intuitively show the performance differences between algorithms, in this paper, in addition to the Wilcoxon rank-sum test results in each benchmark function between TAVOA and comparison algorithm is listed, the statistical comparison of the Wilcoxon rank-sum test in each set of benchmark functions is also listed.

However, none of the metaheuristic optimization algorithms can achieve the best results in all test functions. The main purpose of this paper is to compare TAVOA and AVOA, and verify how much performance TAVOA proposed in this paper can improve. Therefore, if TAVOA achieves better results than AVOA in a function, but is not the best of all comparison algorithms, it is marked with an underline.

### 4.2 Experimental results comparison on the first set functions

In Table 3, the experimental results of TAVOA and six comparison algorithms in the basic unimodal reference function are shown.

**Table 3:** Experimental results of basic unimodal benchmark functions (*f*1−*f*7).

Function		GOA	MPA	PSO	SSA	WOA	AVOA	TAVOA
*f*1	Best	1.62E+000	3.08E-025	6.98E-004	2.09E-008	1.86E-084	0.00E+000	**0.00E+000**
	Worst	6.90E+001	1.55E-022	7.89E-001	9.80E-007	1.36E-072	3.18E-288	**0.00E+000**
	Mean	3.50E+001	4.11E-023	3.55E-002	2.06E-007	5.05E-074	1.06E-289	**0.00E+000**
	Std	1.90E+001	4.30E-023	1.43E-001	2.19E-007	2.47E-073	6.44E-299	**0.00E+000**
*f*2	Best	1.45E+000	7.89E-016	2.86E-003	1.07E-001	9.71E-059	6.29E-190	**3.87E-236**
	Worst	1.19E+002	1.29E-012	2.00E+001	6.08E+000	7.17E-049	3.51E-144	**4.66E-209**
	Mean	2.19E+001	2.23E-013	1.02E+000	1.84E+000	3.02E-050	1.23E-145	**1.63E-210**
	Std	2.62E+001	2.76E-013	4.02E+000	1.59E+000	1.32E-049	6.41E-145	**8.50E-210**
*f*3	Best	8.30E+002	3.84E-008	6.71E+002	3.60E+002	2.53E+004	8.32E-293	**0.00E+000**
	Worst	6.87E+003	1.43E-003	6.84E+003	3.30E+003	8.41E+004	1.66E-203	**0.00E+000**
	Mean	3.34E+003	1.63E-004	1.87E+003	1.59E+003	4.58E+004	5.54E-205	**0.00E+000**
	Std	1.62E+003	2.92E-004	1.74E+003	8.29E+003	1.40E+004	3.03E-204	**0.00E+000**
*f*4	Best	7.74E+000	4.12E-010	4.64E+000	3.13E+000	3.47E+000	3.00E-171	**3.09E-234**
	Worst	2.20E+001	8.54E-009	1.00E+001	2.10E+001	8.84E+001	2.92E-149	**1.96E-201**
	Mean	1.45E+001	3.11E-009	6.77E+000	1.12E+001	4.60E+001	9.92E-151	**6.53E-203**
	Std	3.56E+000	1.56E-009	1.33E+000	4.14E+000	2.95E+001	5.33E-150	**3.57E-202**
*f*5	Best	3.29E+002	2.44E+001	2.82E+001	2.58E+001	2.71E+001	**1.14E-006**	4.79E-005
	Worst	1.65E+004	2.61E+001	9.01E+004	3.31E+003	2.88E+001	**2.86E-004**	2.05E-002
	Mean	3.58E+003	2.54E+001	6.33E+003	4.09E+002	2.81E+001	**7.06E-005**	3.32E-003
	Std	3.39E+003	4.53E-001	2.28E+004	6.58E+002	4.64E-001	**6.58E-005**	4.50E-003
*f*6	Best	8.94E+000	**2.08E-008**	4.62E-004	2.63E-008	6.54E-002	3.98E-008	2.18E-007
	Worst	8.48E+001	**1.05E-007**	1.93E-001	8.17E-007	6.49E-001	1.61E-006	1.06E-005
	Mean	3.67E+001	**4.33E-008**	1.40E-002	1.74E-007	3.34E-001	5.75E-007	3.97E-006
	Std	2.03E+001	**1.87E-008**	3.95E-002	1.90E-007	1.52E-001	3.78E-007	2.94E-006
*f*7	Best	2.19E-002	5.98E-004	2.42E-002	8.27E-002	3.40E-004	6.96E-006	**6.95E-006**
	Worst	8.10E-002	2.72E-003	7.28E-002	3.40E-001	2.02E-002	9.67E-004	**5.96E-004**
	Mean	4.85E-002	1.47E-003	4.75E-002	1.75E-001	3.87E-003	2.21E-004	**1.29E-004**
	Std	1.67E-002	6.22E-004	1.32E-002	7.18E-002	4.13E-003	2.32E-004	**1.32E-004**

As seen from Table 3, the mean value and standard deviation of TAVOA are better than the comparison algorithms on functions *f*1, *f*2, *f*3, *f*4 and *f*7. Especially on functions *f*1 and *f*7, TAVOA finds the best solution to these functions. Although AVOA can also find the best solution on function *f*1, the performance of AVOA is not as stable as TAVOA. In 30 independent experiments, TAVOA can obtain the best solution to function *f*1 every time, but AVOA cannot. On function *f*5, TAVOA achieves only slightly worse results than AVOA. On function *f*6, TAVOA achieves worse results than MPA and AVOA. However, overall, TAVOA performs best among the seven basic unimodal benchmark functions.

**Fig 7:**
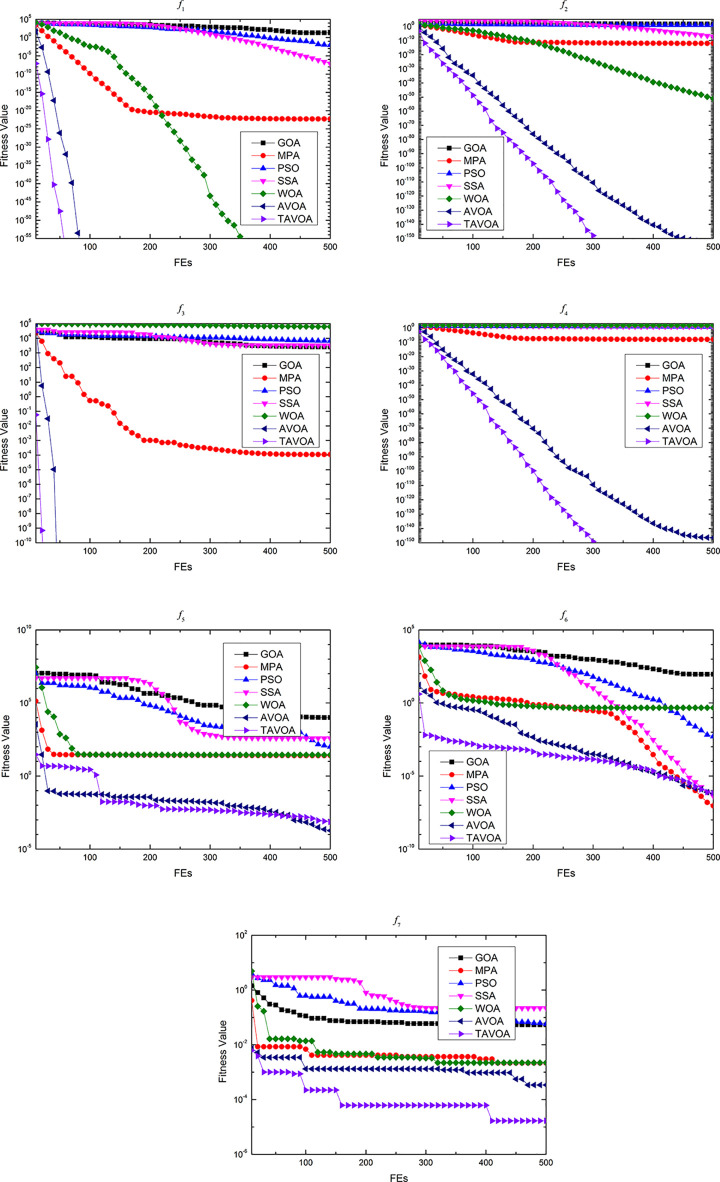
Convergence process on basic unimodal benchmark functions.

To more intuitively analyze the performance of the algorithms and the changes in the solutions obtained by the algorithms during the running of the algorithm, the process of convergence of TAVOA and six comparison functions on 7 basic unimodal benchmark functions is shown in Fig 7.

As seen from Fig 7, TAVOA and AVOA have better performance on functions *f*1, *f*2, *f*3, *f*4, *f*5 and *f*6 than the other five comparison algorithms. In addition, by observing the convergence curves on functions *f*1, *f*2, *f*3, *f*4 and *f*7, it can be found that although the convergence trend of TAVOA is similar to that of AVOA, the results obtained by TAVOA are always better than those obtained by AVOA at the same number of iterations. Especially in functions *f*2 and *f*4, TAVOA can maintain the original convergence speed and obtain better results when AVOA slows down and tends to be stable in the later stage. On function *f*7, it can be seen that TAVOA achieves better results than AVOA at the beginning. When AVOA falls into local optimization in the medium term, TAVOA can jump out of local optimization and achieve better results. In addition, on function *f*5, although TAVOA does not achieve the same good result as AVOA at the beginning, TAVOA can jump out of the local trap in a short time, and the result achieved in the middle is better than AVOA. Similarly, on function *f*6, TAVOA can achieve good results at the beginning and maintain the best results in the early and middle stages. Although it is not as good as MPA and AVOA in the later stage, the difference is not very large.

Therefore, TAVOA shows good performance in these seven basic unimodal functions. Because TAVOA can obtain good solutions in the early and middle stages, it can solve problems with high real-time requirements.

In Table 4, the experimental results of TAVOA and six comparison algorithms in the basic multi-modal reference function are shown.

**Table 4:** Experimental results of basic multi-modal benchmark functions (*f*8−*f*13).

Function		GOA	MPA	PSO	SSA	WOA	AVOA	TAVOA
*f*8	Best	-8.59E+003	-9.81E+003	-9.63E+003	-8.97E+003	-1.26E+004	-1.26E+004	**-1.26E+004**
	Worst	-5.88E+003	-7.98E+003	-7.30E+003	-6.10E+003	-7.47E+003	-8.74E+003	**-1.06E+004**
	Mean	-7.39E+003	-8.88E+003	-8.50E+003	-7.29E+003	-1.03E+004	-1.22E+004	**-1.24E+004**
	Std	6.66E+002	4.84E+002	5.73E+002	7.26E+002	1.82E+003	8.45E+002	**4.78E+002**
*f*9	Best	5.74E+001	**0.00E+000**	2.69E+001	1.69E+001	**0.00E+000**	**0.00E+000**	**0.00E+000**
	Worst	1.60E+002	**0.00E+000**	9.39E+001	1.33E+002	**0.00E+000**	**0.00E+000**	**0.00E+000**
	Mean	9.77E+001	**0.00E+000**	5.32E+001	5.64E+001	**0.00E+000**	**0.00E+000**	**0.00E+000**
	Std	2.92E+001	**0.00E+000**	1.32E+001	2.37E+001	**0.00E+000**	**0.00E+000**	**0.00E+000**
*f*10	Best	3.61E+000	3.49E-013	1.04E-002	5.39E-005	8.88E-016	**8.88E-016**	8.88E-016
	Worst	9.42E+000	3.82E-012	1.78E+000	4.30E+000	1.51E-014	**8.88E-016**	8.88E-016
	Mean	5.46E+000	1.55E-012	5.01E-001	2.48E+000	5.15E-015	**8.88E-016**	8.88E-016
	Std	1.47E+000	8.60E-013	6.50E-001	7.96E-001	3.29E-015	**0.00E+000**	4.01E-031
*f*11	Best	1.00E+000	**0.00E+000**	6.67E-003	8.08E-004	0.00E+000	**0.00E+000**	**0.00E+000**
	Worst	1.31E+000	**0.00E+000**	9.69E-002	5.64E-002	2.36E-001	**0.00E+000**	**0.00E+000**
	Mean	1.15E+000	**0.00E+000**	3.58E-002	1.68E-002	7.87E-003	**0.00E+000**	**0.00E+000**
	Std	7.99E-002	**0.00E+000**	2.41E-002	1.44E-002	4.31E-002	**0.00E+000**	**0.00E+000**
*f*12	Best	4.51E+000	1.10E-009	1.98E-005	1.90E+000	3.92E-003	**5.72E-009**	2.35E-008
	Worst	1.54E+001	1.25E-003	1.45E+000	2.08E+001	1.57E-001	**7.99E-008**	3.23E-007
	Mean	8.98E+000	4.26E-005	1.46E-001	7.69E+000	2.48E-002	**2.77E-008**	1.49E-007
	Std	3.12E+000	2.28E-004	3.04E-001	4.36E+000	3.26E-002	**1.61E-008**	9.04E-008
*f*13	Best	5.60E+000	2.80E-008	1.21E-003	5.79E-002	1.75E-001	**2.54E-009**	1.67E-006
	Worst	8.56E+003	9.94E-002	8.18E-001	5.60E+001	1.59E+000	**2.84E-007**	1.11E-002
	Mean	3.20E+002	8.75E-003	1.07E-001	1.57E+001	5.90E-001	**4.83E-008**	3.89E-004
	Std	1.56E+003	2.06E-002	1.79E-001	1.39E+001	3.09E-001	**5.56E-008**	2.02E-003

As seen from Table 4, the mean value and standard deviation obtained by TAVOA on function *f*8 are better than those of the comparison algorithms. In addition, on function *f*9, TAVOA, like MPA, WOA and AVOA, achieved the global optimal result of the benchmark function in each time of 30 independent experiments. On function *f*11, TAVOA, similar to MPA and AVOA, achieved the globally optimal result of the benchmark function in each time of 30 independent experiments. On function *f*10, although the best value, worst value and mean value obtained by TAVOA are the same as AVOA, TAVOA is not as stable as AVOA. On functions *f*12 and *f*13, the mean value and standard deviation obtained by TAVOA are not as good as AVOA, but they are still better than the remaining five comparison algorithms.

In conclusion, although the performance of TAVOA in the six basic multi-modal functions is not the best, it is not much worse than AVOA.

**Fig 8:**
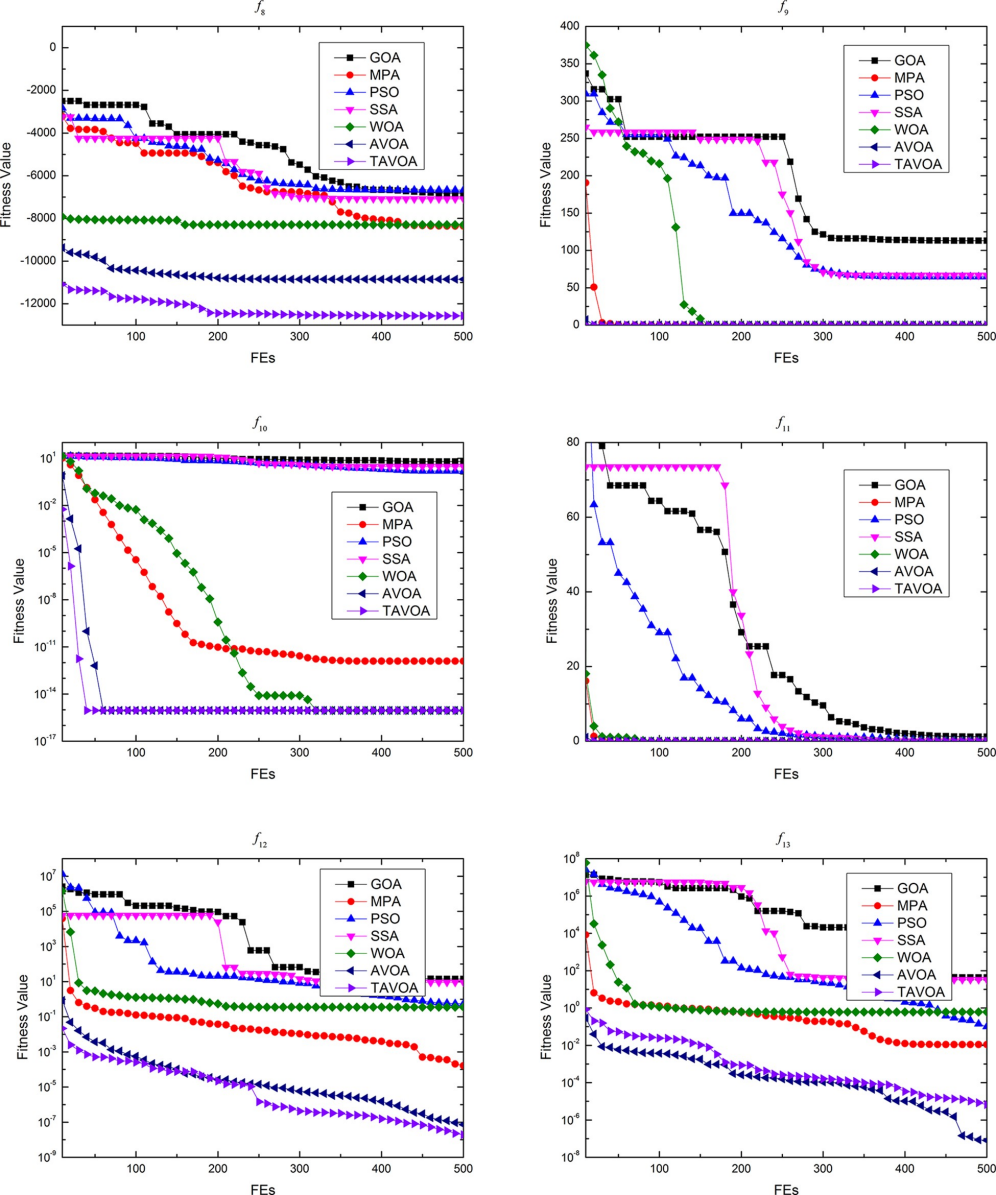
Convergence process on basic multi-modal benchmark functions.

The process of convergence of TAVOA and six comparison functions on 6 basic multi-modal benchmark functions is shown in Fig 8.

As seen from Fig 8, on function *f*8, TAVOA can obtain better results than other algorithms at the beginning and keep a better result all of the time. On function *f*10, although TAVOA is less stable than AVOA in Table 3, it can be seen in Fig 8 that TAVOA can achieve the best results faster than AVOA. In addition, in functions *f*9 and *f*11, TAVOA and AVOA can obtain the best solution of the function at the beginning. Although MPA and SSA can also obtain the best solution of the function in functions *f*9 and *f*11, the speed of MPa and SSA to obtain the best solution is slower than TAVOA and AVOA. Moreover, the stability of SSA in function *f*11 is poor, and the best solution of the function cannot be obtained every time. In function *f*12, the convergence curve of TAVOA decreases rapidly when TAVOA changes from the exploration stage to the exploitation stage, and better results are found. In function *f*13, TAVOA performs worse than AVOA but better than the other five comparison algorithms.

In Table 5, the experimental results of TAVOA and six comparison algorithms on the basic fixed-dimension multi-modal functions are shown.

**Table 5:** Experimental results of basic fixed-dimension multi-modal benchmark functions (*f*14−*f*23).

Function		GOA	MPA	PSO	SSA	WOA	AVOA	TAVOA
*f*14	Best	9.98E-001	9.98E-001	**9.98E-001**	9.98E-001	9.98E-001	9.98E-001	9.98E-001
	Worst	9.98E-001	9.98E-001	**9.98E-001**	2.98E+000	1.08E+001	2.98E+000	2.98E+000
	Mean	9.98E-001	9.98E-001	**9.98E-001**	1.26E+000	2.76E+000	1.26E+000	1.23E+000
	Std	4.12E-016	1.40E-016	**7.14E-017**	5.97E-001	3.36E+000	6.21E-001	5.79E-001
*f*15	Best	6.12E-004	**3.07E-004**	3.08E-004	3.08E-004	3.09E-004	3.08E-004	3.07E-004
	Worst	5.70E-002	**3.07E-004**	2.04E-002	2.04E-002	2.25E-003	1.22E-003	1.22E-003
	Mean	1.10E-002	**3.04E-004**	3.25E-003	1.58E-003	8.18E-004	4.15E-004	3.85E-004
	Std	1.26E-002	**3.78E-015**	6.83E-003	3.56E-003	5.55E-004	1.86E-004	1.75E-004
*f*16	Best	-1.03E+000	-1.03E+000	-1.03E+000	-1.03E+000	-1.03E+000	-1.03E+000	**-1.03E+000**
	Worst	-1.03E+000	-1.03E+000	-1.03E+000	-1.03E+000	-1.03E+000	-1.03E+000	**-1.03E+000**
	Mean	-1.03E+000	-1.03E+000	-1.03E+000	-1.03E+000	-1.03E+000	-1.03E+000	**-1.03E+000**
	Std	2.78E-013	4.83E—016	6.45E-016	2.90E-014	1.47E-009	6.78E-016	**4.40E-016**
*f*17	Best	3.98E-001	**3.98E-001**	**3.98E-001**	3.98E-001	3.98E-001	**3.98E-001**	**3.98E-001**
	Worst	3.98E-001	**3.98E-001**	**3.98E-001**	3.98E-001	3.98E-001	**3.98E-001**	**3.98E-001**
	Mean	3.98E-001	**3.98E-001**	**3.98E-001**	3.98E-001	3.98E-001	**3.98E-001**	**3.98E-001**
	Std	1.88E-013	**0.00E+000**	**0.00E+000**	8.74E-015	1.56E-005	**0.00E+000**	**0.00E+000**
*f*18	Best	3.00E+000	3.00E+000	3.00E+000	3.00E+000	3.00E+000	3.00E+000	**3.00E+000**
	Worst	8.40E+001	3.00E+000	3.00E+000	3.00E+000	3.00E+000	3.00E+000	**3.00E+000**
	Mean	5.70E+000	3.00E+000	3.00E+000	3.00E+000	3.00E+000	3.00E+000	**3.00E+000**
	Std	1.48E+001	2.39E-015	2.00E-015	3.44E-013	8.70E-005	5.01E-006	**0.00E+000**
*f*19	Best	-3.00E-001	-3.00E-001	-3.00E-001	-3.00E-001	-3.00E-001	-3.00E-001	**-3.00E-001**
	Worst	-4.29E+005	-3.00E-001	-3.00E-001	-3.00E-001	-3.00E-001	-3.00E-001	**-3.00E-001**
	Mean	-1.86E-001	-3.00E-001	-3.00E-001	-3.00E-001	-3.00E-001	-3.00E-001	**-3.00E-001**
	Std	1.25E-001	2.26E-016	2.26E-016	2.26E-016	2.26E-016	2.26E-016	**1.13E-016**
*f*20	Best	-3.32E+000	**-3.32E+000**	-3.32E+000	-3.32E+000	-3.32E+000	-3.32E+000	-3.32E+000
	Worst	-3.18E+000	**-3.20E-000**	-3.14E+000	-3.18E+000	-3.04E+000	-3.19E+000	-3.20E-000
	Mean	-3.28E+000	**-3.29E-000**	-3.26E-000	-3.23E+000	-3.24E+000	-3.23E+000	-3.29E-000
	Std	5.96E-002	**1.05E-011**	7.30E-002	5.71E-002	9.94E-002	5.13E-002	5.11E-002
*f*21	Best	-10.1532	-10.1532	-10.1532	-10.1532	-10.1519	-10.1532	**-10.1532**
	Worst	-2.6305	-10.1532	-2.6305	-2.6305	-2.6263	-10.1532	**-10.1532**
	Mean	-6.7229	-10.1532	-6.1468	-7.3929	-8.6090	-10.1532	**-10.1532**
	Std	3.39E+000	1.58E-011	3.3614	3.31E+000	2.61E+000	1.92E-013	**0.00E+000**
*f*22	Best	-10.4028	-10.4028	-10.4028	-10.4028	-10.39919	-10.4028	**-10.4028**
	Worst	-1.83759	-10.4028	-2.75193	-2.75193	-1.83753	-10.4028	**-10.4028**
	Mean	-5.62438	-10.4028	-8.78367	-8.65105	-7.07105	-10.4028	**-10.4028**
	Std	3.35422	6.28E-011	3.11E+000	3.23E+000	3.23E+000	2.20E-013	**0.00E+000**
*f*23	Best	-10.536	-10.536	-10.536	-10.536	-10.535	-10.536	**-10.536**
	Worst	-1.677	-10.536	-2.422	-2.427	-2.420	-10.536	**-10.536**
	Mean	-5.914	-10.536	-7.423	-7.833	-7.393	-10.536	**-10.536**
	Std	3.704	4.64E-011	3.921	3.645	3.47E+000	7.11E-014	**0.00E+000**

As seen from Table 5, the best value, the worst value and the mean value of 30 independent runs obtained by TAVOA on function *f*16 are consistent with the other six comparison algorithms, but the standard deviation of 30 independent runs is smaller than the other six comparison algorithms. Similarly, on functions *f*18 and *f*19, the best value, the worst value and the average value of 30 independent runs obtained by TAVOA are consistent with the remaining five comparison algorithms except for GOA, but the standard deviation of 30 independent runs is smaller than these five comparison algorithms. On functions *f*21, *f*22 and *f*23, TAVOA obtained a better mean value and standard deviation than GOA, PSO, SSA and WOA in 30 independent operations. In addition, on these three functions, the mean value and standard deviation obtained by TAVOA in 30 independent runs are the same as MPA and AVOA, but the standard deviation of 30 independent runs is 0. In other words, TAVOA can obtain the best solution every time on the three functions *f*21, *f*22 and *f*23.

On function *f*17, TAVOA shows the same performance as MPA, PSO and AVOA. The best solution to the function can be obtained every time in 30 independent runs. On functions *f*14, *f*15 and *f*20, although TAVOA does not show the best performance among all algorithms, its mean value and standard deviation obtained in 30 independent runs are better than AVOA. This finding still shows that TAVOA proposed in this paper does improve the performance of AVOA.

In conclusion, TAVOA performs quite well in 10 basic fixed-dimension multi-modal functions and is better than the other 6 algorithms in 7 functions. On the remaining three functions, the performance of TAVOA is also better than that of AVOA.

**Fig 9:**
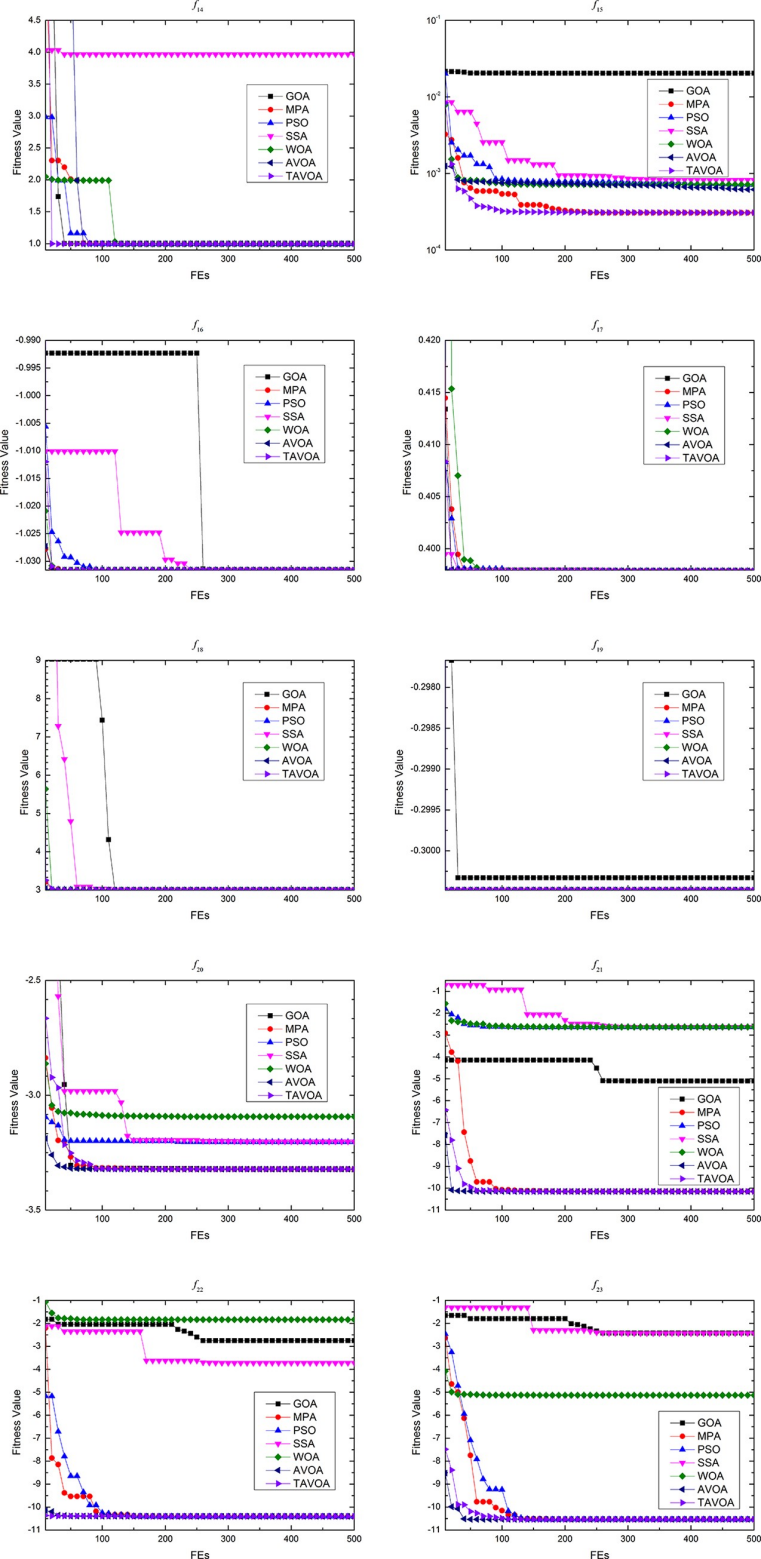
Convergence process on basic fixed-dimension multi-modal benchmark functions.

The process of convergence of TAVOA and six comparison functions on 10 basic multi-modal benchmark functions is shown in Fig 9.

As seen from Fig 9, TAVOA and the six comparison functions perform well on the 10 basic fixed-dimension multi-modal functions, but there is still a certain difference. On functions *f*14, *f*15 and *f*17, TAVOA obtains the best solution faster than the other comparison algorithms. On function *f*19, except for GOA, the other six algorithms can obtain the best solution of the function at the beginning of running. On the six functions of *f*16, *f*17, *f*18, *f*20, *f*21 and *f*23, TAVOA can obtain the best solution in a very early stage, although it is not the fastest. Especially on functions *f*21 and *f*23, it can be seen from Table 5 that TAVOA has higher accuracy and better stability.

In Table 6, the Wilcoxon rank-sum test with 5% on each function of 23 basic benchmark functions and the statistical results of the Wilcoxon rank-sum test are shown.

**Table 6:** The results of the Wilcoxon rank-sum statistical test with 5% among TAVOA and the 6 compared algorithms on the 23 basic benchmark functions.

Type	Function	GOA	MPA	PSO	SSA	WOA	AVOA
**Unimodal benchmark function**	*f*1	1.21E-012+	1.21E-012+	1.21E-012+	1.21E-012+	1.21E-012+	2.79E-003+
	*f*2	3.02E-011+	3.02E-011+	3.02E-011+	3.02E-011+	3.02E-011+	3.02E-011+
	*f*3	1.21E-012+	1.21E-012+	1.21E-012+	1.21E-012+	1.21E-012+	1.21E-012+
	*f*4	3.02E-011+	3.02E-011+	3.02E-011+	3.02E-011+	3.02E-011+	3.02E-011+
	*f*5	3.02E-011+	3.02E-011+	3.02E-011+	3.02E-011+	3.02E-011+	1.61E-010-
	*f*6	3.02E-011+	3.02E-011-	3.02E-011+	1.21E-010-	3.02E-011+	5.09E-008-
	*f*7	3.02E-011+	3.02E-011+	3.02E-011+	3.02E-011+	7.39E-011+	1.15E-001 =
**Multi-modal benchmark function**	*f*8	1.43E-011+	1.43E-011+	1.43E-011+	1.43E-011+	1.28E-007+	2.57E-002+
	*f*9	1.21E-012+	NaN =	1.21E-012+	1.21E-012+	NaN =	NaN =
	*f*10	1.21E-012+	1.21E-012+	1.21E-012+	1.21E-012+	1.36E-004+	1.69E-014-
	*f*11	1.21E-012+	NaN =	1.21E-012+	1.21E-012+	3.34E-001 =	NaN =
	*f*12	3.02E-011+	1.07E-007+	3.02E-011+	3.02E-011+	3.02E-011+	1.69E-009-
	*f*13	3.02E-011+	3.18E-001 =	4.08E-011+	3.02E-011+	3.02E-011+	3.02E-011-
**Fixed-dimension multi-modal benchmark function**	*f*14	2.67E-007-	8.56E-008-	8.54E-008-	1.29E-007+	1.88E-008+	1.26E-007+
	*f*15	1.21E-010+	3.02E-011-	1.63E-005+	4.57E-009+	5.09E-006+	4.92E-001 =
	*f*16	1.21E-012+	1.19E-013+	1.18E-013+	1.21E-012+	1.21E-012+	4.16E-014+
	*f*17	1.21E-012+	NaN =	NaN =	1.10E-012+	NaN =	NaN =
	*f*18	1.21E-012+	1.11E-012+	9.34E-013+	2.05E-005+	1.21E-012+	1.21E-012+
	*f*19	1.10E-012+	1.69E-014+	1.69E-014+	1.69E-014+	1.69E-014+	1.69E-014+
	*f*20	5.50E-001 =	2.92E-004-	1.13E-001 =	1.61E-004+	3.93E-001 =	9.51E-004+
	*f*21	6.41E-001 =	1.21E-012+	3.46E-001 =	3.47E-001 =	1.21E-012+	1.20E-012+
	*f*22	1.21E-012+	1.21E-012+	3.87E-013+	1.21E-012+	1.21E-012+	1.19E-012+
	*f*23	5.89E-002 =	1.21E-012+	1.56E-001 =	5.89E-002 =	1.21E-012+	1.18E-012+
+/ = /-		19/3/1	15/4/4	18/4/1	20/2/1	19/4/0	13/5/5

It seen from Table 6 that TAVOA outperforms GOA, MPA, PSO, SSA, WOA and AVOA on 19, 15, 18, 20, 19, 13 basic benchmark functions respectively. Moreover, TAVOA only performs worse than GOA, MPA, PSO, SSA, WOA and AVOA on 1, 4, 1, 1, 0, 5 functions respectively.

In summary, among the 23 basic benchmark functions, TAVOA performs better than the other 6 comparison algorithms. Especially on the 10 basic fixed-dimension multi-modal functions, TAVOA shows better performance than the original AVOA. Besides, TAVOA is significantly better than AVOA on 8 functions out of the 10 basic fixed-dimension multi-modal functions. In addition, TAVOA is inferior to AVOA on only five functions in the basic unimodal benchmark function and basic multi-modal benchmark function. Therefore, overall, TAVOA greatly improves the performance of AVOA.

### 4.3 Experimental results comparison on the second set functions

To further and more comprehensively test the ability of TAVOA and comparison algorithm to solve continuous optimization problems, a more professional test benchmark function set CEC 2013 is used. At the same time, because CEC 2013 is more complex, in addition to the **boldface** and underline, the ranking of the algorithm in a test benchmark function and the average ranking in a certain type of benchmark function are given to more intuitively show how much the performance improves with TAVOA.

In Table 7, the experimental results of TAVOA and six comparison algorithms on the CEC 2013 unimodal functions are shown.

**Table 7:** Experimental results of CEC 2013 unimodal benchmark functions (f24−f28).

Function		GOA	MPA	PSO	SSA	WOA	AVOA	TAVOA
*f*24	Best	-1.40E+003	-1.40E+003	-1.40E+003	-1.40E+003	-1.40E+003	**-1.40E+003**	**-1.40E+003**
	Worst	-8.92E+002	-1.40E+003	8.37E+003	-1.40E+003	-1.40E+003	**-1.40E+003**	**-1.40E+003**
	Mean	-1.35E+003	-1.40E+003	1.74E+003	-1.40E+003	-1.40E+003	**-1.40E+003**	**-1.40E+003**
	Std	1.60E+002	4.67E-012	2.47E+003	9.06E-011	3.49E-006	**0.00E+000**	**0.00E+000**
	Rank	6	3	7	4	5	**1**	**1**
*f*25	Best	3.31E+005	-4.83E+002	4.69E+003	4.71E+003	**1.52E+003**	3.11E+004	1.43E+004
	Worst	1.98E+007	7.89E+004	2.57E+007	5.99E+004	**1.27E+003**	6.86E+003	1.10E+005
	Mean	3.16E+006	1.71E+004	7.99E+006	3.36E+004	**5.64E+003**	2.15E+005	5.03E+004
	Std	5.26E+006	1.83E+004	7.06E+003	1.67E+004	**2.59E+003**	1.44E+005	2.51E+004
	Rank	6	2	7	3	**1**	5	4
*f*26	Best	1.06E+007	**6.22E+003**	1.63E+008	7.27E+005	2.70E+003	4.47E+006	5.97E+005
	Worst	1.72E+010	**2.37E+008**	1.58E+002	2.27E+008	1.27E+010	2.07E+009	2.70E+009
	Mean	2.84E+009	**2.13E+007**	8.17E+010	3.96E+007	3.48E+009	3.06E+008	2.70E+008
	Std	3.91E+009	**4.40E+007**	2.86E+011	5.07E+007	3.60E+009	4.30E+008	5.01E+008
	Rank	5	**1**	7	2	6	4	3
*f*27	Best	-5.70E+002	-1.10E+003	-1.10E+003	**-1.10E+003**	-4.85E+002	-1.10E+003	-1.10E+003
	Worst	1.45E+004	-1.10E+003	7.84E+004	**-1.10E+003**	1.06E+004	-1.10E+003	-1.10E+003
	Mean	5.46E+003	-1.10E+003	5.97E+003	**-1.10E+003**	2.63E+003	-1.10E+003	-1.10E+003
	Std	4.65E+003	5.95E-007	1.50E+004	**6.30E-010**	2.29E+003	1.46E-007	3.44E+000
	Rank	6	3	7	**1**	5	2	4
*f*28	Best	-1.00E+003	-1.00E+003	-1.00E+003	-1.00E+003	-1.00E+003	-1.00E+003	**-1.00E+003**
	Worst	1.43E+003	-1.00E+003	7.67E+003	-1.00E+003	-1.00E+003	-1.00E+003	**-1.00E+003**
	Mean	-4.66E+002	-1.00E+003	4.53E+002	-1.00E+003	-1.00E+003	-1.00E+003	**-1.00E+003**
	Std	6.69E+002	2.62E-007	1.83E+003	3.04E-005	2.35E-003	7.23E-007	**2.14E-007**
	Rank	6	2	7	4	5	3	**1**
Average rank		5.8	2.2	7	2.8	4.4	3	2.6

As seen from Table 7, on function *f*24, TAVOA, like AVOA, obtains the best solution of the function every time in 30 independent runs. On function *f*28, although the best value, worst value and mean value obtained by TAVOA in 30 independent runs are the same as MPA, SSA, WOA and AVOA, the standard deviation obtained by TAVOA is smaller. On functions *f*25 and *f*26, although the performance of TAVOA is not the best among all comparison algorithms, the mean value and standard deviation of TAVOA are better than those of AVOA in 30 independent runs. On function *f*27, TAVOA obtains the same best value, worst value and standard deviation as MPA, SSA, WOA and AVOA in 30 independent operations, but the standard deviation is large. Therefore, the accuracy of TAVOA on function *f*27 is not as good as that of the four comparison algorithms. In addition, according to the average ranking in Table 7, the average ranking of TAVOA is 2.6, which is worse than 2.2 of MPA and 2.8 of SSA but better than 3 of AVOA. Therefore, the performance of TAVOA on CEC 2013 unimodal benchmark functions is better than AVOA. Although the average ranking of TAVOA is lower than MPA and WOA, TAVOA outperforms MPA and WOA in the performance of functions *f*24 and *f*28. Moreover, both MPA and WOA outperform TAVOA on only one function.

**Fig 10:**
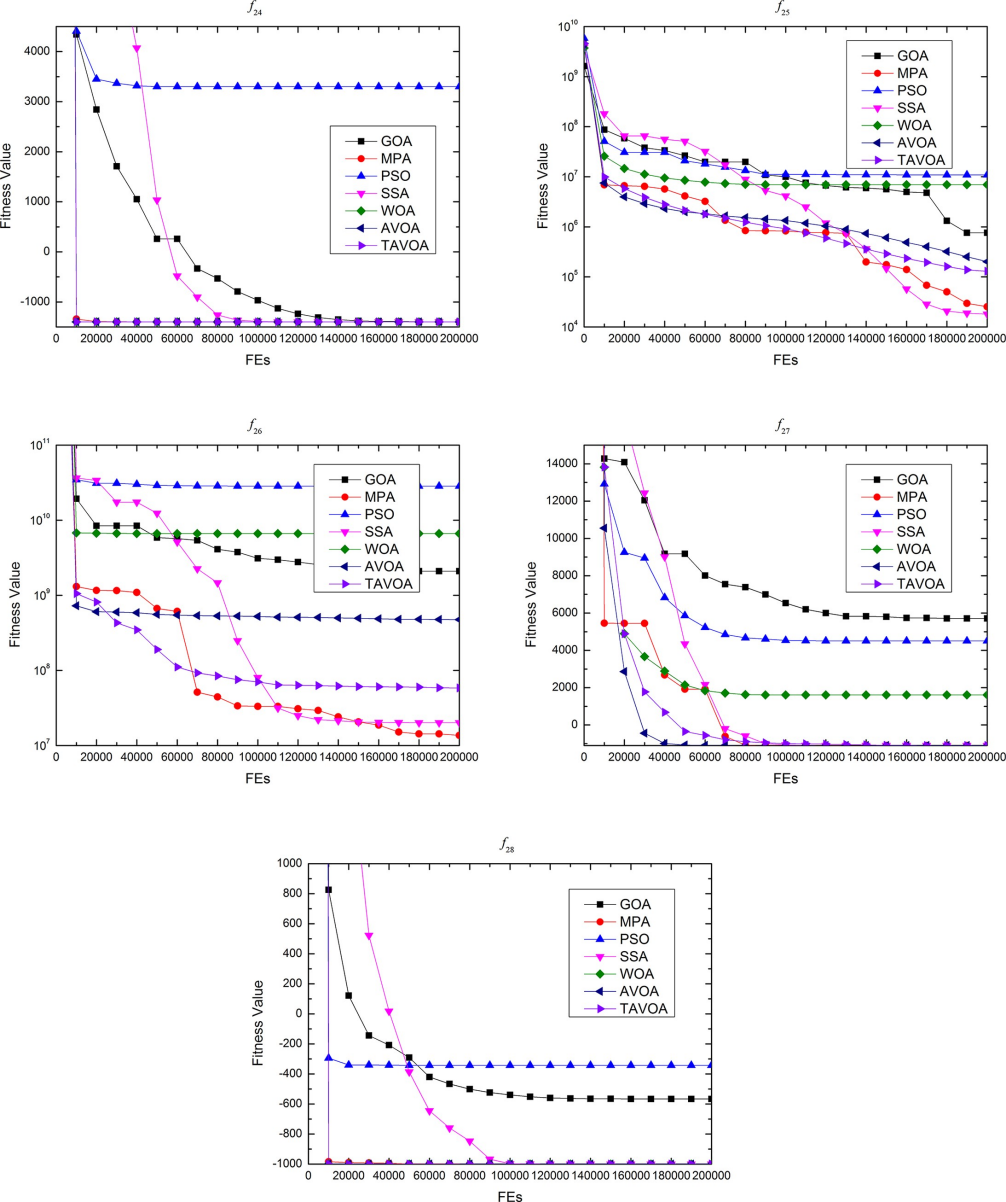
Convergence process on CEC 2013 unimodal benchmark functions.

The process of convergence of TAVOA and six comparison functions on 5 CEC 2013 unimodal benchmark functions is shown in Fig 10.

As seen from Fig 10, TAVOA can obtain the best solution of the function in the early stage on functions *f*24 and *f*28. On functions *f*25 and *f*26, although TAVOA is inferior to MPA and SSA in the final performance, it can be found from the convergence curve that the feasible solution obtained by TAVOA in the early stage is better than MPA and SSA. In other words, if a problem requires high real-time performance, TAVOA shows better performance than MPA and SSA. In addition, on functions *f*25 and *f*26, although the feasible solution obtained by TAVOA at the beginning is not as good as AVOA, TAVOA can make full use of exploration and exploitation abilities in a very short time to find a better feasible solution than AVOA. In function *f*27, TAVOA performs worse than AVOA. AVOA can quickly find the best solution of the function, while TAVOA needs to gradually find the best solution of the function in the exploitation stage. The reason is that when we designed TAVOA, we balanced its exploration and exploitation capabilities, in such a way that although TAVOA cannot obtain a good feasible solution at the beginning, its balanced exploration ability and exploitation ability will exist through the whole algorithm’s run to improve on the performance of AVOA.

Since there are 15 multi-modal functions of CEC 2013, they are disassembled into two groups for a more convenient display. In Table 8, the experimental results of TAVOA and six comparison algorithms on the CEC 2013 multimodal reference function (*f*29−*f*36) are shown.

**Table 8:** Experimental results of CEC 2013 basic multimodal benchmark functions (*f*29−*f*36).

Function		GOA	MPA	PSO	SSA	WOA	AVOA	TAVOA
*f*29	Best	-8.85E+002	-9.00E+002	-8.45E+002	-9.00E+002	-8.93E+002	-9.00E+002	**-9.00E+002**
	Worst	-7.90E+002	-8.15E+002	5.73E+001	-8.74E+002	-7.65E+002	-8.31E+002	**-8.71E+002**
	Mean	-8.38E+002	-8.93E+002	-6.51E+002	-8.90E+002	-8.46E+002	-8.87E+002	**-8.96E+002**
	Std	2.72E+001	1.60E+001	2.04E+002	6.70E+000	3.34E+001	1.96E+001	**1.17E+001**
	Rank	6	2	7	3	5	4	**1**
*f*30	Best	-7.90E+002	-7.80E+002	-7.76E+002	**-7.94E+002**	-6.95E+002	-7.03E+002	-7.24E+002
	Worst	-6.90E+002	-7.34E+002	-5.20E+002	**-7.37E+002**	3.34E+003	-5.54E+002	-5.26E+002
	Mean	-7.56E+002	-7.57E+002	-6.88E+002	**-7.75E+002**	-4.89E+002	-6.43E+002	-6.55E+002
	Std	2.61E+001	1.11E+001	6.63E+001	**1.46E-001**	7.26E+002	3.01E+001	4.87E+001
	Rank	3	2	4	**1**	7	6	5
*f*31	Best	-6.79E+002	-6.79E+002	-6.79E+002	-6.79E+002	-6.79E+002	-6.79E+002	**-6.79E+002**
	Worst	-6.79E+002	-6.79E+002	-6.79E+002	-6.79E+002	-6.79E+002	-6.79E+002	**-6.79E+002**
	Mean	-6.79E+002	-6.79E+002	-6.79E+002	-6.79E+002	-6.79E+002	-6.79E+002	**-6.79E+002**
	Std	4.75E-002	5.75E-002	4.61E-002	4.67E-002	3.86E-002	6.18E-002	**4.31E-002**
	Rank	5	6	3	4	2	7	**1**
*f*32	Best	**-5.87E+002**	-5.74E+002	-5.88E+002	-5.88E+002	-5.73E+002	-5.78E+002	-5.73E+002
	Worst	**-5.75E+002**	-5.69E+002	-5.67E+002	-5.75E+002	-5.61E+002	-5.64E+002	-5.62E+002
	Mean	**-5.83E+002**	-5.70E+002	-5.69E+002	-5.82E+002	-5.66E+002	-5.69E+002	-5.70E+002
	Std	**3.32E+000**	3.58E+000	2.76E+000	3.76E+000	3.25E+000	3.42E+000	2.81E+000
	Rank	**1**	4	5	2	7	6	3
*f*33	Best	-5.00E+002	-5.00E+002	-4.45E+002	-5.00E+002	-5.00E+002	-5.00E+002	**-5.00E+002**
	Worst	-1.92E+002	-5.00E+002	1.28E+003	-5.00E+002	-4.98E+002	-4.99E+002	**-5.00E+002**
	Mean	-4.23E+002	-5.00E+002	2.07E+001	-5.00E+002	-4.99E+002	-5.00E+002	**-5.00E+002**
	Std	7.53E+001	9.07E-002	4.83E+002	9.53E-002	5.07E-001	2.26E-001	**2.03E-002**
	Rank	6	2	7	3	5	4	**1**
*f*34	Best	-3.29E+002	**-4.00E+002**	-3.82E+002	-3.65E+002	-2.61E+002	-3.99E+002	-4.00E+002
	Worst	-2.38E+002	**-4.00E+002**	-2.93E+002	-2.64E+002	1.35E+002	-3.87E+002	-3.92E+002
	Mean	-2.94E+002	**-4.00E+022**	-3.46E+002	-3.16E+002	-1.63E+001	-3.95E+002	-3.98E+002
	Std	2.67E+001	**2.42E-011**	2.77E+001	2.68E+001	8.40E+001	3.26E+000	2.04E+000
	Rank	6	**1**	4	5	7	3	2
*f*35	Best	-2.55E+002	-4.49E+002	-2.34E+002	**-2.55E+002**	-8.18E+001	-1.00E+002	-2.07E+001
	Worst	-1.35E+002	-1.69E+002	-9.61E+001	**-1.62E+002**	5.13E+002	3.74E+002	2.61E+002
	Mean	-2.08E+002	-2.10E+002	-1.84E+002	**-2.16E+002**	2.09E+002	1.51E+002	1.00E+002
	Std	3.10E+001	2.04E+001	3.37E+001	**2.19E+001**	1.24E+002	1.13E+002	8.01E+001
	Rank	2	3	4	**1**	7	6	5
*f*36	Best	-1.10E+002	-1.03E+002	-1.02E+002	**-1.26E+002**	7.67E+001	5.34E+001	3.70E+001
	Worst	4.73E+001	1.92E+001	1.71E+001	**-1.63E+001**	3.27E+002	2.51E+002	2.67E+002
	Mean	-3.64E+001	-3.79E+001	-3.78E+001	**-4.62E+001**	2.13E+002	1.39E+002	1.31E+002
	Std	3.86E+001	-3.79E+001	3.04E+001	**3.35E+001**	6.46E+001	5.41E+001	5.61E+001
	Rank	4	2	3	**1**	7	6	5
Average rank		4.125	2.75	4.625	**2.5**	5.875	5.25	2.875

As seen from Table 8, on function *f*29, although the best value obtained by TAVOA in 30 independent runs is the same as MPA, SSA and AVOA, the obtained worst value, mean value and standard deviation are better than the other six comparison algorithms. On function *f*31, the best value, worst value and mean value obtained by TAVOA and the six comparison algorithms in 30 independent runs are the same, but the standard deviation obtained by TAVOA is smaller than that of the other six comparison algorithms. On function *f*33, the best values of the other six algorithms are the same in 30 independent runs except for PSO. Although the mean value obtained by TAVOA in 30 independent runs is the same as MPA, SSA and AVOA on function *f*33, the standard deviation obtained by TAVOA is the smallest of all comparison algorithms. Although TAVOA does not perform best in functions *f*30, *f*32, *f*34, *f*35 and *f*36, TAVOA outperforms AVOA. Especially on function *f*32, TAVOA greatly exceeds AVOA in ranking. In terms of average ranking, TAVOA has also overtaken AVOA to a great extent. Although TAVOA lags behind SSA and MPA in the average ranking, it exceeds GOA and PSO, which are better than AVOA. Therefore, it can be seen that in the first group of multi-modal functions of CEC 2013, TAVOA not only has good performance but also greatly improves the performance of AVOA.

**Fig 11:**
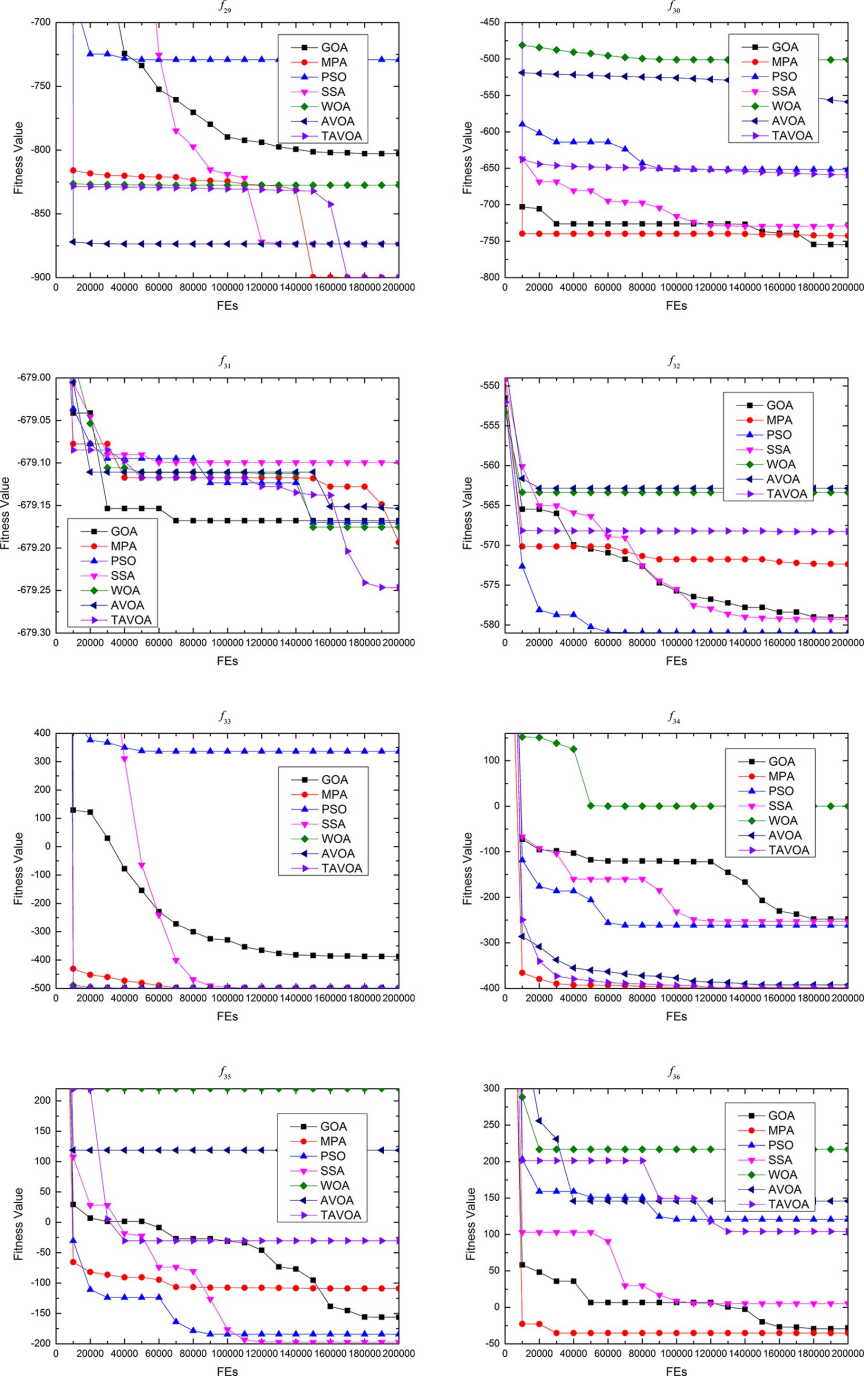
Convergence process on CEC 2013 multi-modal benchmark functions (*f*29−*f*36).

The process of convergence of TAVOA and six comparison functions on 8 CEC 2013 multi-modal benchmark functions (*f*29−*f*36) is shown in Fig 11.

As seen from Fig 11, on function *f*33, although TAVOA and AVOA can obtain good feasible solutions in the shortest time, it can be found that TAVOA has better accuracy and stability than AVOA in Table 8. On functions *f*29 and *f*36, although the feasible solution obtained by TAVOA is not as good as AVOA in the early and middle stages, TAVOA can jump out of the local trap and obtain a better feasible solution in the later stage. In combination with Table 8, it can be found that the standard deviation obtained by TAVOA in 30 independent runs is the smallest. In other words, the ability of TAVOA to jump out of local traps on functions *f*29 and *f*36 is not accidental. The reason is that TAVOA adds a certain exploration ability in the later stage and assigns different weights to different vultures. TAVOA is not the best on functions *f*30, *f*32 and *f*34, but TAVOA obtains better feasible solutions than AVOA. Although the feasible solution obtained by TAVOA on function *f*34 is not as good as AVOA at the beginning, TAVOA can surpass AVOA to obtain a better feasible solution in the early stage of the algorithm. On function *f*35, the feasible solution obtained by TAVOA at the beginning is not as good as AVOA and falls into local optimization.

However, TAVOA can quickly jump out of the local trap and obtain a better feasible solution than AVOA. On function *f*31, similarly, although the feasible solution obtained by TAVOA is not as good as other comparison algorithms at the beginning, TAVOA can continue to find better feasible solutions with the running of the algorithm, and TAVOA can still maintain good exploitation ability in the later stage.

In Table 9, the experimental results of TAVOA and six comparison algorithms on CEC 2013 multi-modal benchmark functions (*f*37−*f*43) are shown.

**Table 9:** Experimental results of CEC 2013 basic multimodal benchmark functions (*f*37−*f*43).

Function		GOA	MPA	PSO	SSA	WOA	AVOA	TAVOA
*f*37	Best	2.76E+003	**-9.87E+001**	7.97E+002	2.15E+003	2.39E+003	-2.55E+001	6.40E+001
	Worst	4.21E+003	**1.48E+002**	3.00E+003	4.01E+003	5.34E+003	1.55E+003	1.06E+003
	Mean	3.56E+003	**-4.20E+001**	1.83E+003	3.17E+003	4.05E+003	6.33E+002	4.53E+002
	Std	4.63E+002	**6.65E+001**	5.79E+002	4.60E+002	7.64E+002	4.26E+002	2.81E+002
	Rank	4	**1**	2	3	4	7	5
*f*38	Best	2.54E+003	1.88E+003	2.47E+003	**1.88E+003**	3.08E+003	3.49E+003	3.31E+003
	Worst	5.02E+003	4.39E+003	5.85E+003	**4.76E+003**	6.57E+003	5.77E+003	6.04E+003
	Mean	3.48E+003	3.37E+003	3.68E+003	**3.23E+003**	5.18E+003	4.90E+003	4.68E+003
	Std	5.97E+002	5.76E+002	7.94E+002	**6.67E+002**	8.17E+002	6.07E+002	6.58E+002
	Rank	3	2	4	**1**	7	6	5
*f*39	Best	2.00E+002	2.00E+002	2.01E+002	2.00E+002	2.01E+002	2.00E+002	**2.00E+002**
	Worst	2.01E+002	2.01E+002	2.02E+002	2.01E+002	2.02E+002	2.02E+002	**2.02E+002**
	Mean	2.00E+002	2.01E+002	2.01E+002	2.00E+002	2.01E+002	2.01E+002	**2.00E+002**
	Std	1.70E-001	2.89E-001	3.41E-001	1.55E-001	3.80E-001	4.44E-001	**1.23E-001**
	Rank	3	4	5	2	6	7	**1**
*f*40	Best	3.76E+002	**3.30E+002**	3.37E+002	3.83E+002	6.05E+002	3.35E+002	3.39E+002
	Worst	4.40E+002	**3.31E+002**	6.02E+002	5.31E+002	1.02E+003	3.56E+002	3.93E+002
	Mean	4.08E+002	**3.31E+002**	3.69E+002	4.26E+002	8.40E+002	3.40E+002	3.58E+002
	Std	1.66E+001	**1.71E-001**	6.13E+001	3.43E+001	1.01E+002	4.77E+000	1.27E+001
	Rank	5	**1**	4	6	7	2	3
*f*41	Best	4.68E+002	4.89E+002	5.05E+002	**4.75E+002**	7.22E+002	6.82E+002	6.13E+002
	Worst	5.55E+002	6.18E+002	6.84E+002	**5.89E+002**	1.24E+003	1.07E+003	1.02E+003
	Mean	5.05E+002	5.39E+002	5.85E+002	**5.14E+002**	9.13E+002	9.10E+002	8.10E+002
	Std	2.06E+001	2.59E+001	3.01E+001	**2.33E+001**	1.14E+002	1.13E+002	1.07E+002
	Rank	2	3	4	**1**	7	6	5
*f*42	Best	5.03E+002	**5.01E+002**	5.02E+002	5.02E+002	5.18E+002	5.02E+002	5.03E+002
	Worst	5.16E+002	**5.02E+002**	1.92E+004	5.08E+002	5.70E+002	5.07E+002	5.07E+002
	Mean	5.07E+002	**5.01E+002**	2.16E+003	5.05E+002	5.44E+002	5.04E+002	5.04E+002
	Std	3.98E+000	**3.20E-001**	4.09E+003	1.35E+000	1.36E+001	1.24E+000	1.00E+000
	Rank	5	**1**	7	4	6	3	2
*f*43	Best	6.10E+002	**6.09E+002**	6.09E+002	6.08E+002	6.13E+002	6.12E+002	6.12E+002
	Worst	6.15E+002	**6.12E+002**	6.12E+002	6.12E+002	6.15E+002	6.15E+002	6.15E+002
	Mean	6.14E+002	**6.10E+002**	6.11E+002	6.10E+002	6.14E+002	6.14E+002	6.14E+002
	Std	1.88E+000	**6.47E-001**	8.08E-001	8.27E-001	3.88E-001	9.34E-001	7.10E-001
	Rank	7	**1**	3	2	4	6	5
Average rank		4.1	1.9	4.1	2.7	5.9	5.3	3.7

As seen from Table 9, on function *f*39, although the best value and mean value obtained by TAVOA in 30 independent runs are the same as GOA and SSA, the standard deviation obtained by TAVOA is smaller than all comparison algorithms. In particular, from the perspective of ranking, TAVOA has greatly improved AVOA from the original seventh to the first. On the five functions *f*37, *f*38, *f*41, *f*42 and *f*43, although the mean value and standard deviation obtained by TAVOA according to the results of 30 independent runs are not the best of all algorithms, the mean values obtained by TAVOA on the three functions *f*37, *f*38 and *f*41 are better than AVOA. In addition, on functions *f*42 and *f*43, although the mean value obtained by TAVOA according to the results of 30 independent runs is the same as AVOA, the standard deviation obtained by TAVOA is smaller than AVOA. Therefore, in the above five functions, TAVOA shows better performance than AVOA in terms of mean value and stability. Although the mean value and standard deviation obtained by TAVOA in 30 independent runs on function *f*40 are not as good as AVOA, from the perspective of ranking, TAVOA is only one place behind AVOA, and the gap is not very large. In conclusion, in the second group of CEC 2013 multi-modal functions, the overall performance of TAVOA is still better than that of AVOA. Similarly, TAVOA lags behind MPA and SSA in the average ranking but exceeds GOA and PSO, which are better than AVOA. Therefore, it can be seen that in the second group of CEC 2013 multi-modal functions, TAVOA also greatly improves the performance of AVOA.

**Fig 12:**
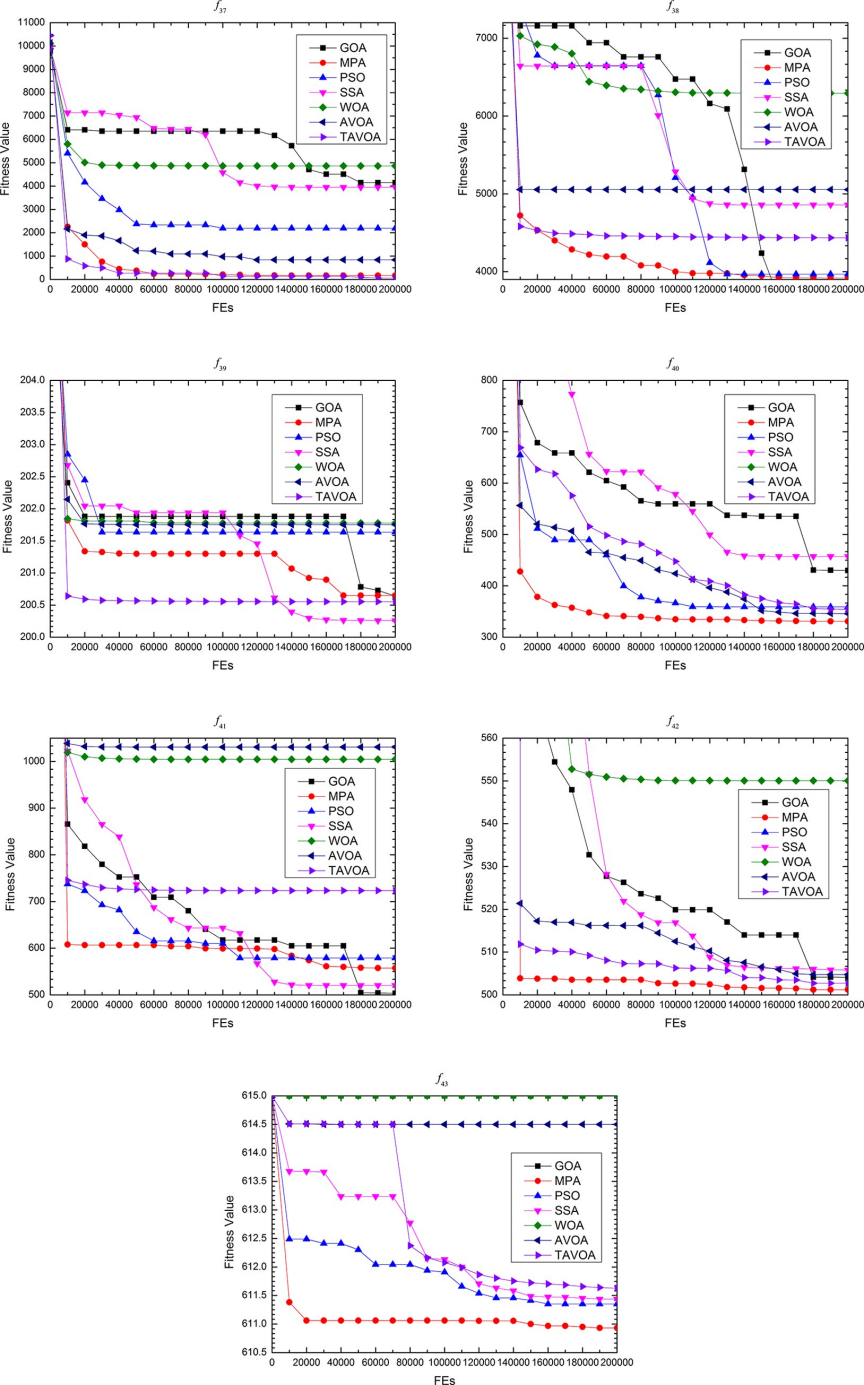
Convergence process on CEC 2013 multi-modal benchmark functions (*f*37−*f*43).

The process of convergence of TAVOA and six comparison functions on 7 CEC 2013 multi-modal benchmark functions (*f*37−*f*43) is shown in Fig 12.

As seen from Fig 12, on the three functions *f*37, *f*38, *f*39 and *f*41, TAVOA can obtain a better feasible solution and converge at the beginning. In addition, on the three functions, the feasible solution obtained by TAVOA is significantly better than AVOA. On the function *f*42, TAVOA can also obtain better feasible solutions at the beginning, and TAVOA continues to obtain better feasible solutions with the running of the algorithm. Moreover, TAVOA always obtains better feasible solutions on function *f*42 than AVOA. However, the feasible solution obtained by TAVOA is not as good as AVOA on function *f*40. Although TAVOA continues to obtain better feasible solutions in the whole stage of algorithm running and even obtains as good feasible solutions as AVOA in the middle of the algorithm, it is still not as good as AVOA in the later stage. Nevertheless, Fig 12 shows that the gap between AVOA and TAVOA on function *f*40 is very small. This finding can also be seen from the function ranking in Table 9. Therefore, on the second group of CEC 2013 multi-modal functions, TAVOA still shows good performance. TAVOA is better than AVOA in both real-time and precision requirements.

In Table 10, the experimental results of TAVOA and six comparison algorithms on CEC 2013 composition benchmark functions (*f*44−*f*51) are shown.

**Table 10:** Experimental results of CEC 2013 composition benchmark functions (*f*44−*f*51).

Function		GOA	MPA	PSO	SSA	WOA	AVOA	TAVOA
*f*44	Best	8.00E+002	8.00E+002	9.00E+002	9.00E+002	9.00E+002	1.00E+003	**9.00E+002**
	Worst	1.14E+003	1.14E+003	1.78E+003	1.14E+003	1.14E+003	1.14E+003	**1.14E+003**
	Mean	1.06E+002	1.05E+003	1.21E+003	1.05E+003	1.07E+003	1.09E+003	**9.97E+002**
	Std	8.54E+001	1.21E+002	2.08E+002	7.95E+001	7.14E+001	7.04E+001	**7.66E+001**
	Rank	4	2	7	3	5	6	**1**
*f*45	Best	4.14E+003	8.05E+002	1.74E+003	3.50E+003	4.31E+003	9.57E+002	**9.97E+002**
	Worst	7.45E+003	1.17E+003	4.49E+003	5.20E+003	8.14E+003	1.85E+003	**1.84E+003**
	Mean	5.41E+003	1.06E+003	3.80E+003	4.30E+003	6.03E+003	1.38E+003	**1.03E+003**
	Std	8.35E+002	9.46E+001	7.09E+002	5.04E+002	1.00E+003	2.51E+002	**2.41E+002**
	Rank	6	2	4	5	7	3	**1**
*f*46	Best	**3.60E+003**	3.00E+003	3.68E+003	2.71E+003	5.15E+003	5.49E+003	5.66E+003
	Worst	**5.62E+003**	5.73E+003	6.39E+003	6.64E+003	8.93E+003	8.40E+003	8.40E+003
	Mean	**4.39E+003**	4.40E+003	4.89E+003	4.64E+003	6.93E+003	7.02E+003	6.85E+003
	Std	**5.02E+002**	6.48E+002	6.80E+002	7.92E+002	1.09E+003	7.77E+002	8.62E+002
	Rank	**1**	2	4	3	7	6	5
*f*47	Best	1.25E+003	1.23E+003	1.25E+003	**1.22E+003**	1.28E+003	1.27E+003	1.27E+003
	Worst	1.28E+003	1.38E+003	1.29E+003	**1.28E+003**	1.31E+003	1.32E+003	1.31E+003
	Mean	1.26E+003	1.36E+003	1.27E+003	**1.25E+003**	1.30E+003	1.29E+003	1.29E+003
	Std	1.00E+003	1.06E+001	1.05E+000	**1.20E+003**	8.56E+000	1.13E+001	8.59E+000
	Rank	2	7	3	**1**	6	5	4
*f*48	Best	1.36E+003	1.37E+003	1.36E+003	1.37E+003	1.38E+003	1.38E+003	**1.38E+003**
	Worst	1.39E+003	1.42E+003	1.42E+003	1.39E+003	1.43E+003	1.43E+003	**1.41E+003**
	Mean	1.39E+003	1.41E+003	1.40E+003	1.39E+003	1.41E+003	1.40E+003	**1.39E+003**
	Std	6.72E+000	7.14E+000	1.19E+001	6.37E+000	1.25E+001	1.18E+001	**5.45E+000**
	Rank	3	6	5	2	7	4	**1**
*f*49	Best	1.40E+003	1.40E+003	1.40E+003	1.40E+003	1.40E+003	1.40E+003	**1.40E+003**
	Worst	1.57E+003	1.40E+003	1.57E+003	1.40E+003	1.60E+003	1.40E+003	**1.40E+003**
	Mean	1.52E+003	1.40E+003	1.54E+003	1.40E+003	1.48E+003	1.40E+003	**1.40E+003**
	Std	6.22E+001	9.05E-003	3.87E+001	4.91E-003	9.46E+001	1.57E-002	**4.40E-003**
	Rank	6	3	7	2	5	4	**1**
*f*50	Best	**1.86E+003**	2.01E+003	1.96E+003	1.85E+003	2.40E+003	2.20E+003	2.25E+003
	Worst	**2.15E+003**	2.34E+003	2.32E+003	2.25E+003	2.71E+003	2.59E+003	2.63E+003
	Mean	**2.04E+003**	2.18E+003	2.14E+003	2.09E+003	2.56E+003	2.49E+003	2.47E+003
	Std	**7.46E+001**	7.42E+001	6.74E+001	9.97E+001	7.52E+001	9.14E+001	9.86E+001
	Rank	**1**	4	3	2	7	6	5
*f*51	Best	1.50E+003	**1.50E+003**	1.70E+003	1.50E+003	2.89E+003	1.50E+003	1.50E+003
	Worst	3.01E+003	**1.70E+003**	3.92E+003	2.78E+003	7.10E+003	6.39E+003	5.87E+003
	Mean	1.87E+003	**1.67E+003**	3.64E+003	1.76E+003	5.17E+003	3.34E+003	3.60E+003
	Std	4.58E+002	**6.94E+001**	5.20E+002	2.79E+002	1.13E+003	1.69E+003	1.62E+003
	Rank	3	**1**	6	2	7	4	5
Average rank		3.25	3.375	4.875	2.5	6.375	4.75	2.875

As seen from Table 10, on functions *f*44 and *f*45, in 30 independent runs, TAVOA obtains a better mean value and standard deviation than the other six comparison algorithms. Especially on the function *f*45, from the perspective of ranking, TAVOA has greatly improved the ranking of AVOA, from the original sixth place to the first place.

On function *f*48, TAVOA also performs best. Although in 30 independent runs, the best value and the worst value obtained by TAVOA are worse than GOA, and the mean value is the same as GOA, and the standard deviation obtained by TAVOA is smaller than GOA. This finding shows that the best value obtained by GOA and the worst value obtained by TAVOA are accidental and that TAVOA is more stable than GOA. Similarly, on function *f*49, in 30 independent runs, TAVOA obtains the same best value, worst value and mean value as MPA, SSA and AVOA, but the standard deviation obtained by TAVOA is smaller than other comparison algorithms. In addition, TAVOA performs better than AVOA in functions *f*46, *f*47 and *f*50, although it is not the best. At the same time, on function *f*51, the performance of TAVOA is not as good as AVOA, but it does not differ much. TAVOA still outperforms PSO and WOA on function *f*51.

In summary, TAVOA performs quite well in CEC 2013 composition benchmark functions. It performs best on four functions and performs better than AVOA on three functions. Similarly, TAVOA lags behind SSA in the average ranking, but exceeds GOA and MPA,which are better than AVOA. Therefore, among the eight combined benchmark functions of CEC 2013, TAVOA also greatly improves the performance of AVOA.

**Fig 13:**
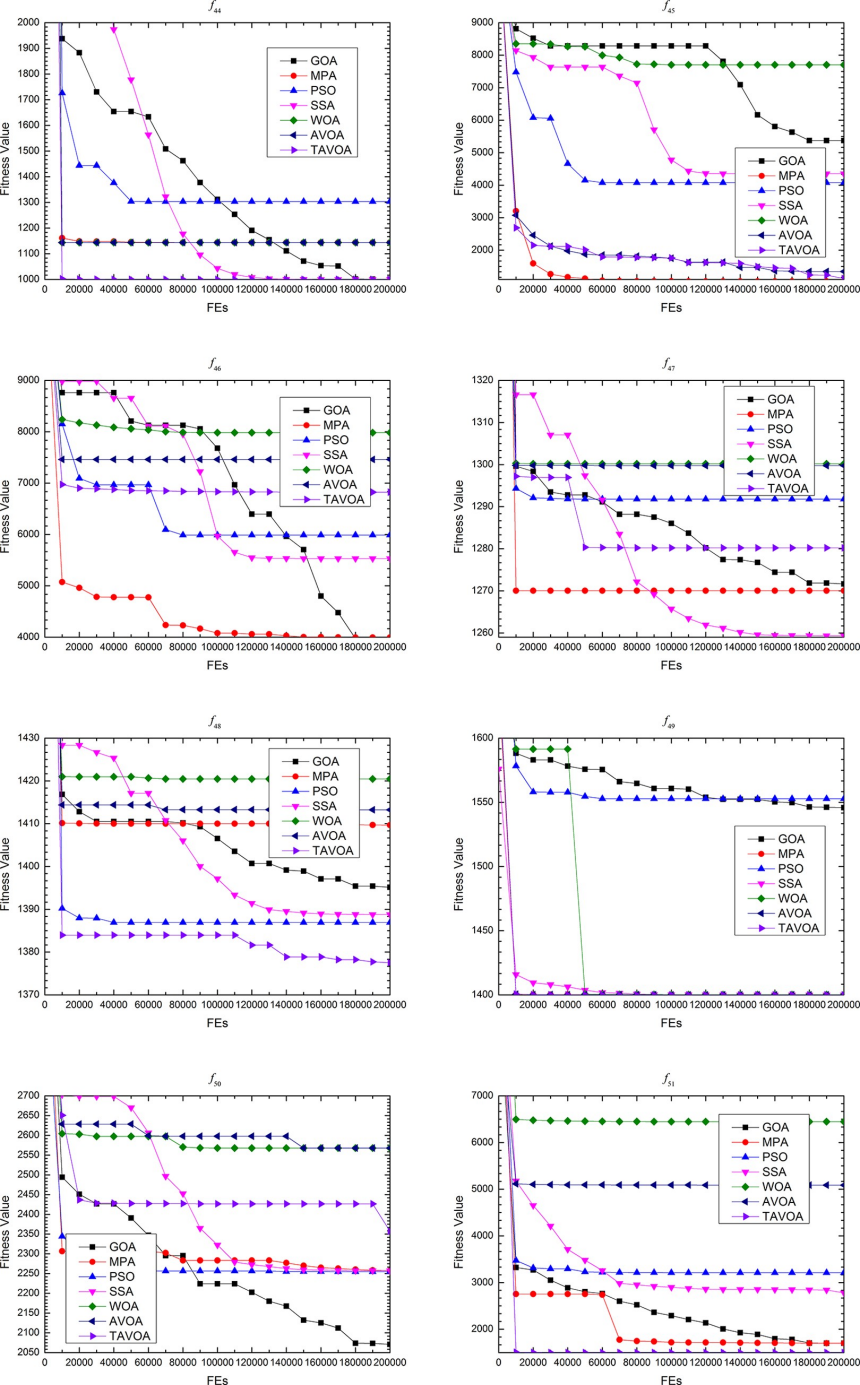
Convergence process on CEC 2013 composition benchmark functions.

The process of convergence of TAVOA and six comparison functions on 8 CEC 2013 composition benchmark functions (*f*44−*f*51) is shown in Fig 13.

As seen from Fig 13, TAVOA can quickly obtain better feasible solutions and converge on functions *f*44, *f*49 and *f*51. On function *f*45, by comparing the convergence curves of TAVOA and AVOA, it can be found that TAVOA obtains a better feasible solution than AVOA at the beginning. With the continuous run of the algorithm, TAVOA and AVOA continue to obtain a better feasible solution. However, in the later stage of the algorithm, AVOA falls into the local optimal solution, and TAVOA can jump out of the local trap and obtain a better feasible solution than AVOA. On function *f*46, AVOA converged at the beginning, while TAVOA not only obtain a better feasible solution than AVOA at the beginning but also did not converge in the early stage, but continued to find a better feasible solution. Although the feasible solution obtained in this process has not improved much compared with the beginning, it also proves that TAVOA still has a certain exploitation ability in the early stage. On the function *f*47, AVOA also converged at the beginning, while TAVOA not only obtained a better feasible solution than AVOA at the beginning, but also used the exploration ability to jump out of the local trap and obtain a better feasible solution in the early stage. On function *f*48, TAVOA obtains a better feasible solution than other comparison algorithms at the beginning. In addition, when other comparison algorithms gradually stabilize and fall into the local optimal solution, TAVOA can still use the exploration ability in the medium stage and use the exploitation ability in the later stage to find a better feasible solution. Similarly, on function *f*50, although TAVOA obtained a better feasible solution than AVOA at the beginning and fell into the local optimization, it can be found from the convergence curve of TAVOA that TAVOA still has certain exploration ability in the later stage, which can help TAVOA jump out of the local trap and obtain a better feasible solution.

In summary, on the CEC 2013 composition benchmark functions, we can fully understand that TAVOA has always guaranteed sufficient exploration ability and exploitation ability during the running of the whole algorithm. TAVOA not only has a certain exploitation ability in the early stage but also has a certain exploration ability in the late stage to ensure that TAVOA can jump out of the local trap in the late stage while obtaining a better solution in the early stage.

In Table 11, the Wilcoxon rank-sum test with 5% on each function of 28 CEC 2013 benchmark functions and the statistical results of the Wilcoxon rank-sum test are shown.

**Table 11:** The results Wilcoxon rank-sum statistical test with 5% among TAVOA and the 6 compared algorithms on the 28 CEC 2013 benchmark functions.

Type	Function	GOA	MPA	PSO	SSA	WOA	AVOA
**Unimodal benchmark function**	*f*24	1.96E-009+	2.00E-010+	2.13E-006+	2.53E-011+	2.53E-011+	NaN =
	*f*25	8.64E-009+	2.93E-006-	6.68E-011+	1.49E-002-	6.68E-011-	7.44E-009+
	*f*26	3.31E-005+	2.71E-006-	3.77E-010+	2.63E-004-	1.81E-009+	6.46E-001 =
	*f*27	8.64E-009+	6.68E-011-	1.90E-003-	6.68E-011-	6.68E-011+	6.68E-011-
	*f*28	8.64E-009+	6.13E-001 =	1.12E-009+	6.68E-011+	6.68E-011+	1.16E-006+
**Multi-modal benchmark function**	*f*29	1.16E-007+	4.05E-001 =	6.68E-011+	2.00E-006+	1.17E-007+	6.74E-004+
	*f*30	1.85E-008-	6.68E-011-	1.10E-001 =	6.68E-011-	1.43E-002+	4.52E-002+
	*f*31	1.56E-002+	1.59E-001 =	3.31E-001 =	5.07E-002 =	9.44E-002 =	7.15E-001 =
	*f*32	8.64E-009-	7.55E-010+	6.68E-011+	6.68E-011-	2.88E-003+	3.96E-001 =
	*f*33	4.54E-006+	1.27E-005+	6.68E-011+	8.22E-011+	6.68E-011+	3.63E-001 =
	*f*34	8.07E-009+	2.14E-008-	6.38E-011+	6.38E-011+	6.38E-011+	6.73E-005+
	*f*35	8.64E-009-	6.68E-011-	6.68E-011-	6.68E-011-	2.97E-004+	4.75E-002+
	*f*36	9.82E-009-	6.68E-011-	6.68E-011-	6.68E-011-	1.57E-005+	5.08E-001 =
	*f*37	8.64E-009-	1.01E-010-	1.12E-010-	6.68E-011-	6.68E-011-	1.37E-001 =
	*f*38	1.41E-006-	8.16E-009-	1.10E-005-	6.78E-009-	1.00E-002+	1.59E-001 =
	*f*39	6.52E-009+	1.69E-008+	7.69E-006+	3.77E-010+	2.60E-003+	1.78E-001 =
	*f*40	2.38E-008+	6.68E-011-	9.44E-004+	1.24E-010+	6.68E-011+	4.67E-009-
	*f*41	8.64E-009-	7.41E-011-	1.01E-010-	6.68E-011-	1.63E-003+	1.31E-002+
	*f*42	1.82E-001 =	6.68E-011-	7.02E-005+	7.26E-001 =	6.68E-011+	1.83E-001 =
	*f*43	5.09E-003+	6.66E-011-	1.01E-010-	1.01E-010-	2.87E-004-	1.05E-002+
**Composition benchmark function**	*f*44	5.95E-001 =	1.48E-002+	1.45E-001 =	5.47E-003+	7.26E-003+	4.37E-001 =
	*f*45	8.64E-009+	5.61E-010+	9.11E-011+	6.68E-011+	6.68E-011+	4.32E-001 =
	*f*46	8.64E-009-	8.22E-011-	2.79E-010-	3.41E-010-	8.95E-001 =	3.71E-001 =
	*f*47	1.44E-008-	9.11E-011+	6.78E-009-	9.11E-011-	1.55E-002+	6.69E-001 =
	*f*48	9.11E-008+	5.13E-009+	1.31E-002+	3.77E-010+	8.89E-006+	5.08E-001 =
	*f*49	1.12E-008+	7.55E-010+	6.68E-011+	1.84E-002+	6.68E-011+	8.16E-009+
	*f*50	8.64E-009-	1.87E-010-	8.22E-011-	7.41E-011-	4.77E-004+	2.59E-001 =
	*f*51	5.73E-002 =	1.84E-001 =	8.27E-002 =	5.50E-002 =	2.33E-004+	1.63E-001 =
+/ = /-		15/3/10	9/4/15	14/4/10	11/3/14	23/2/3	9/17/2

It seen from Table 11 that TAVOA better than GOA, MPA, PSO, SSA, WOA and AVOA on 15, 9, 14, 11, 23, 9 CEC 2013 benchmark functions respectively. Moreover, TAVOA performs worse than GOA, MPA, PSO, SSA, WOA and AVOA on 10, 15, 10, 14, 3, 2 functions respectively.

In general, although the performance of AVOA on 28 CEC 2013 benchmark functions is not as outstanding as that on 23 basic benchmark functions, we can still see that the performance of TAVOA is still better than GOA, PSO, WOA and AVOA. In particular, the performance of TAVOA on 9 CEC 2013 functions is significantly better than AVOA, while it is weaker than AVOA only on 2 CEC 2013 functions. Therefore, TAVOA still improves the performance of AVOA on the CEC 2013 functions.

### 4.4 Real-word engineering applications

In order to further verify the effectiveness and practicability of TAVOA proposed in this paper, three common mechanical design problems are selected to verify the performance of TAVOA, and the results will also be compared with AVOA and five other state-of-the-art metaheuristic algorithms mentioned in the previous section. The three real-world engineering problems are welded beam design, compression/tension spring design, and pressure vessel design [55]. However, engineering problems in the real world are often accompanied by natural constraints. In order to meet these constraints in the process of program implementation, this paper adopts the method of external penalty approach mechanism. In the external penalty approach mechanism, if a metaheuristic algorithm violates any constraints, its fitness value will be subject to high penalties.

In addition, in order to make the experiment fairer and more persuasive, the common parameters used in all experiments are consistent with the previous section. That is, the experimental results of all algorithms are obtained when the population size is 30 and the number of independent runs is 30. In addition, because the common real-world engineering problems are more similar to the 23 basic benchmark functions, the maximum number of iterations of all metaheuristic algorithms involved in this paper is 500 for the three real-world engineering problems in this section.

In this paper, in addition to using the best value, mean value, worst value and standard deviation of the 30 independent running results of each metaheuristic optimization algorithm to evaluate the performance of the metaheuristic algorithm, its convergence process diagram is also used to observe the convergence speed of each metaheuristic algorithm.

#### 4.4.1 The welded beam design problem

**Fig 14:**
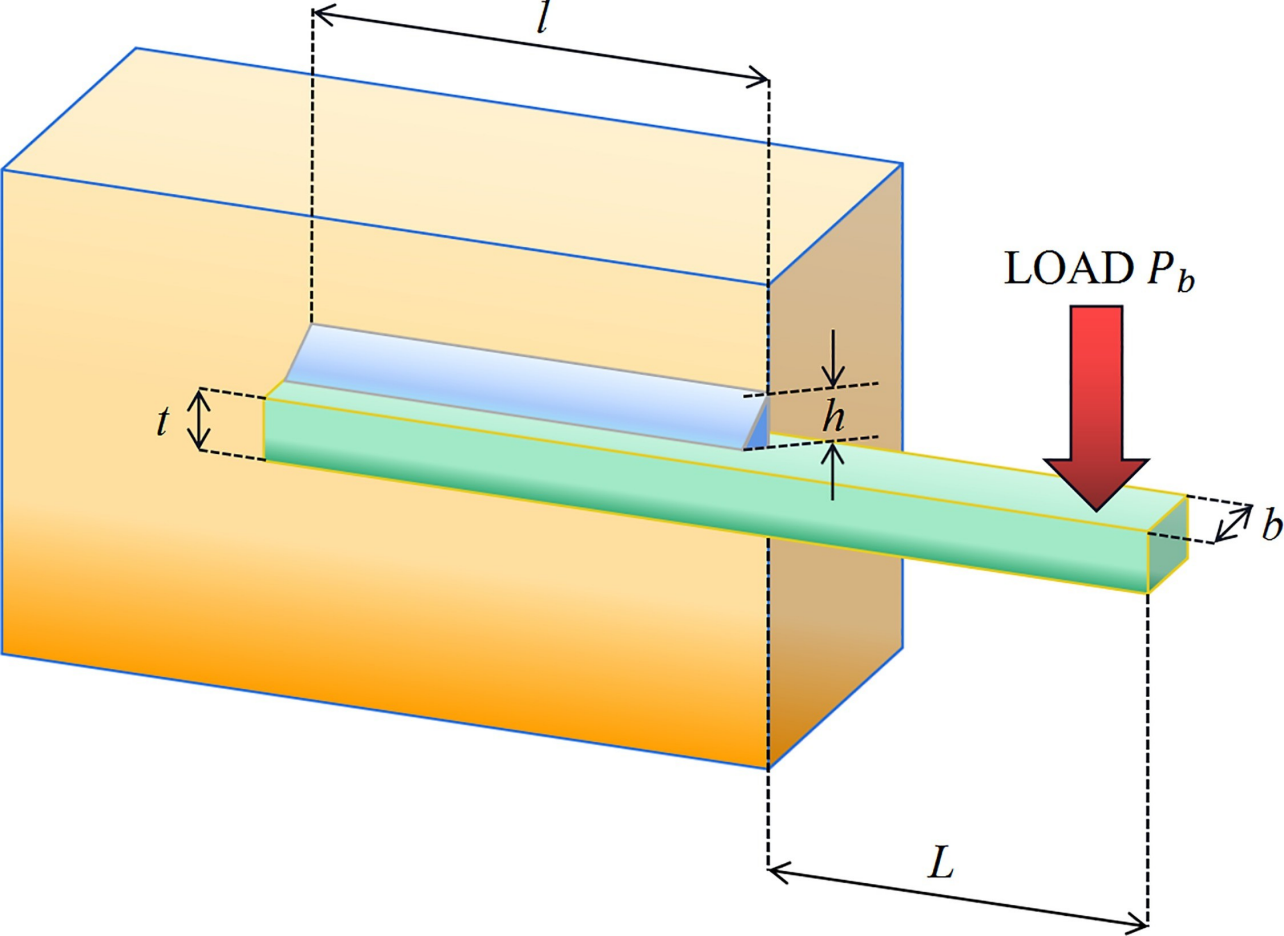
3D schematic view of the welded beam design problem.

As shown in Fig 14, the main goal to be solved in the welded beam design problem is how to select the thickness of weld (*h*), the length of the attached bar (*l*), the height of the bar (*t*), and the thickness of the bar (*b*) to minimize the cost of a welded beam [56].

According to the limitations of physical laws, the welded beam design problem also includes bending stress in beam, buckling load on the bar, shear stress, and the deflection. If the variables to be optimized are considered as *X* = [*x*_1_, *x*_2_, *x*_3_, *x*_4_] = [*h*, *l*, *t*, *b*], the problem is described as Eq (27), and its constraints are defined as Eq (28).

$$f(X)=1.10471x_{1}^{2}x_{2}+0.04811x_{3}x_{4}(14.0+x_{2})$$ (27)

$$\left\{ \begin{array}{c} g_{1}(X)=\tau (X)-\tau_{max}\leq 0\\ g_{2}(X)=\sigma (X)-\sigma_{max}\leq 0\\ g_{3}(X)=\delta (X)-\delta_{max}\leq 0\\ g_{4}(X)=x_{1}-x_{4}\leq 0\\ g_{5}(X)=P-P_{c}(X)\leq 0\\ g_{6}(X)=0.125-x_{1}\leq 0\\ g_{7}(X)=1.10471x_{1}^{2}+0.04811x_{3}x_{4}(14.0+x_{2})-5\leq 0 \end{array}\right.$$ (28)

where:

$$\tau (X)=\sqrt{{\left(\tau^{\prime }\right)}^{2}+2\tau^{\prime }\tau^{\prime \prime }\frac{x_{2}}{2R}+{\left(\tau^{\prime \prime }\right)}^{2}},\tau^{\prime }=\frac{P}{\sqrt{2}x_{1}x_{2}},\tau^{\prime \prime }=\frac{MR}{J}$$

$$R=\sqrt{\frac{x_{2}^{2}}{4}+{\left(\frac{x_{1}+x_{3}}{2}\right)}^{2}}$$

$$J=2\left\{\sqrt{2}x_{1}x_{2}[\frac{x_{2}^{2}}{12}+{\left(\frac{x_{1}+x_{3}}{2}\right)}^{2}]\right\}$$

$$P_{c}(X)=\frac{4.013E\sqrt{\frac{x_{3}^{2}x_{4}^{6}}{36}}}{L^{2}}\left(1-\frac{x_{3}}{2L}\sqrt{\frac{E}{4G}}\right)$$

$$\sigma (X)=\frac{6PL}{x_{4}x_{3}^{2}},\delta (X)=\frac{4PL^{3}}{Ex_{3}^{2}x_{4}},\tau_{max}=13600psi,\sigma_{max}=30000psi$$

$$P=6000lb,L=14in,E=30\times 10^{6}psi,G=12\times 10^{6}psi,\delta_{max}=0.25in$$

$$0.1\leq x_{1},x_{4}\leq 2.0,0.1\leq x_{2},x_{3}\leq 10$$

The best value, the worst value, mean value and standard deviation obtained by TAVOA and comparison algorithms in the welded beam design problem are shown in Table 12.

**Table 12:** Statistical results of the welded beam design problem.

Algorithm	Best	Mean	Worst	SD
GOA	1.697591862	2.751736464	4.603852566	0.913214103
MPA	1.695245232	**1.695248373**	**1.695264079**	**3.60328E-06**
PSO	**1.695244929**	1.696711264	1.729417061	0.006348954
SSA	1.704307936	1.808793916	2.044134761	0.097684495
WOA	1.805787349	434.8904015	12971.97296	2367.877003
AVOA	1.696468635	1.733036108	1.833292101	0.040227062
TAVOA	1.695663888	1.724550104	1.81576298	0.031850462

As seen from Table 12 that PSO achieves the best results in the welded beam design problem, but MPA is the most stable. Although TAVOA is not as good as PSO and MPA, it is better than the other five comparison algorithms. In addition, the performance of TAVOA is better than that of AVOA in terms of the best value, the worst value and stability.

Table 13 lists the values of each variable when TAVOA and the comparison algorithms achieve the optimum cost in solving the welded beam design problem.

**Table 13:** Best results of the welded beam design problem.

Algorithms	Optimum variables				Optimum cost
	*h*	*l*	*t*	*b*	
GOA	0.20363	3.2899	9.04	0.20571	1.697591862
MPA	0.20574	3.253	9.0366	0.20573	1.695245232
PSO	0.20573	3.253	9.0366	0.20573	**1.695244929**
SSA	0.19684	3.4189	9.0366	0.20573	1.704307936
WOA	0.21492	3.6592	8.8156	0.21618	1.805787349
AVOA	0.20446	3.276	9.0366	0.20573	1.696468635
TAVOA	0.20531	3.2606	9.0365	0.20573	1.695663888

**Fig 15:**
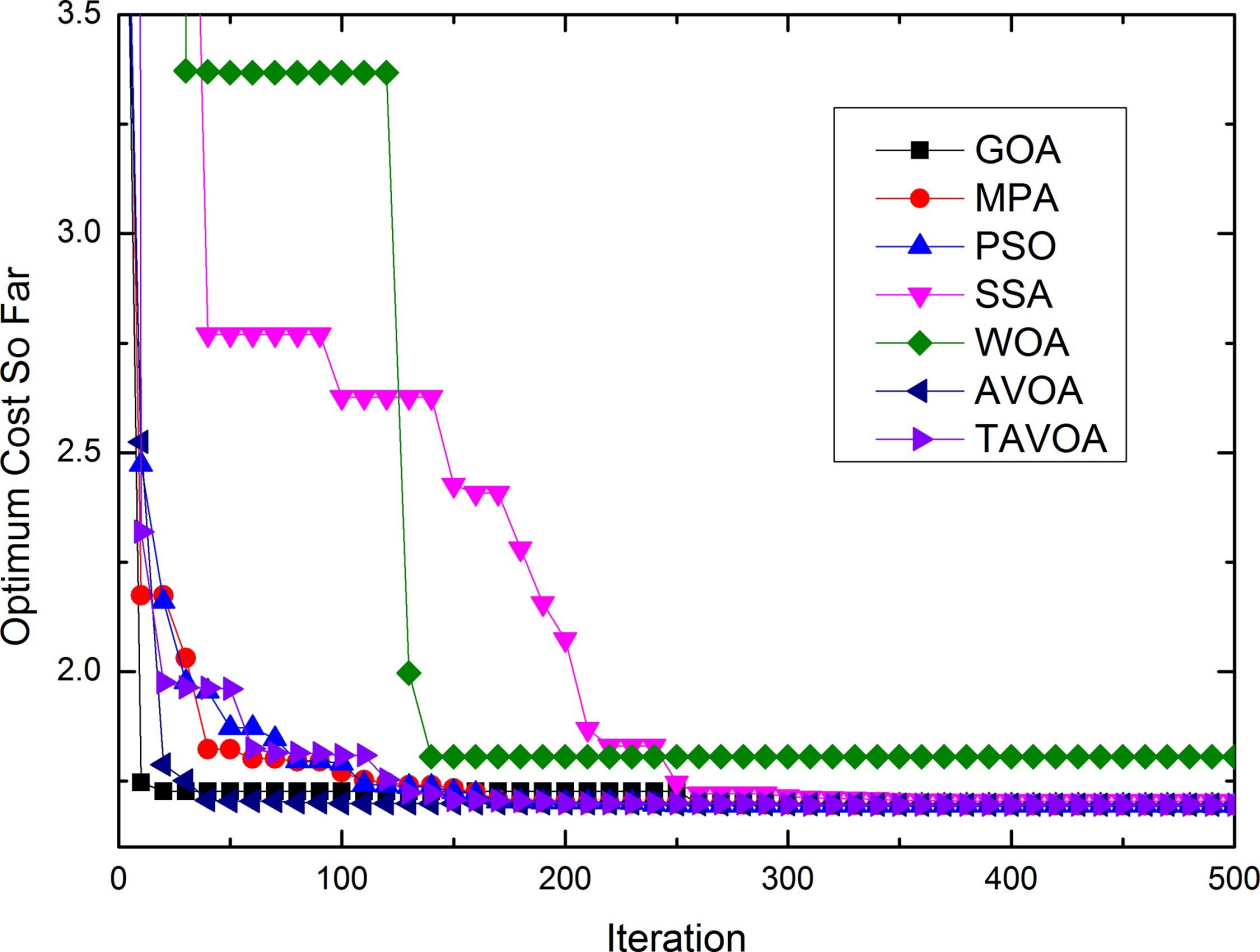
Convergence process in the welded beam design problem.

The convergence curves of TAVOA and comparison algorithms when obtaining the best value in the welded beam design problem are shown in Fig 15.

As seen from Fig 15, TAVOA converges slightly faster than MPA and PSO. Although AVOA converges very early, AVOA falls into the locally optimal solution. In addition, GOA gets a better solution very early, but it still falls into locally trap. Although GOA jumps out of the current locally trap at 250 iterations, the solution obtained by GOA is still not as good as TAVOA.

#### 4.4.2 The compression/tension spring design problem

**Fig 16:**
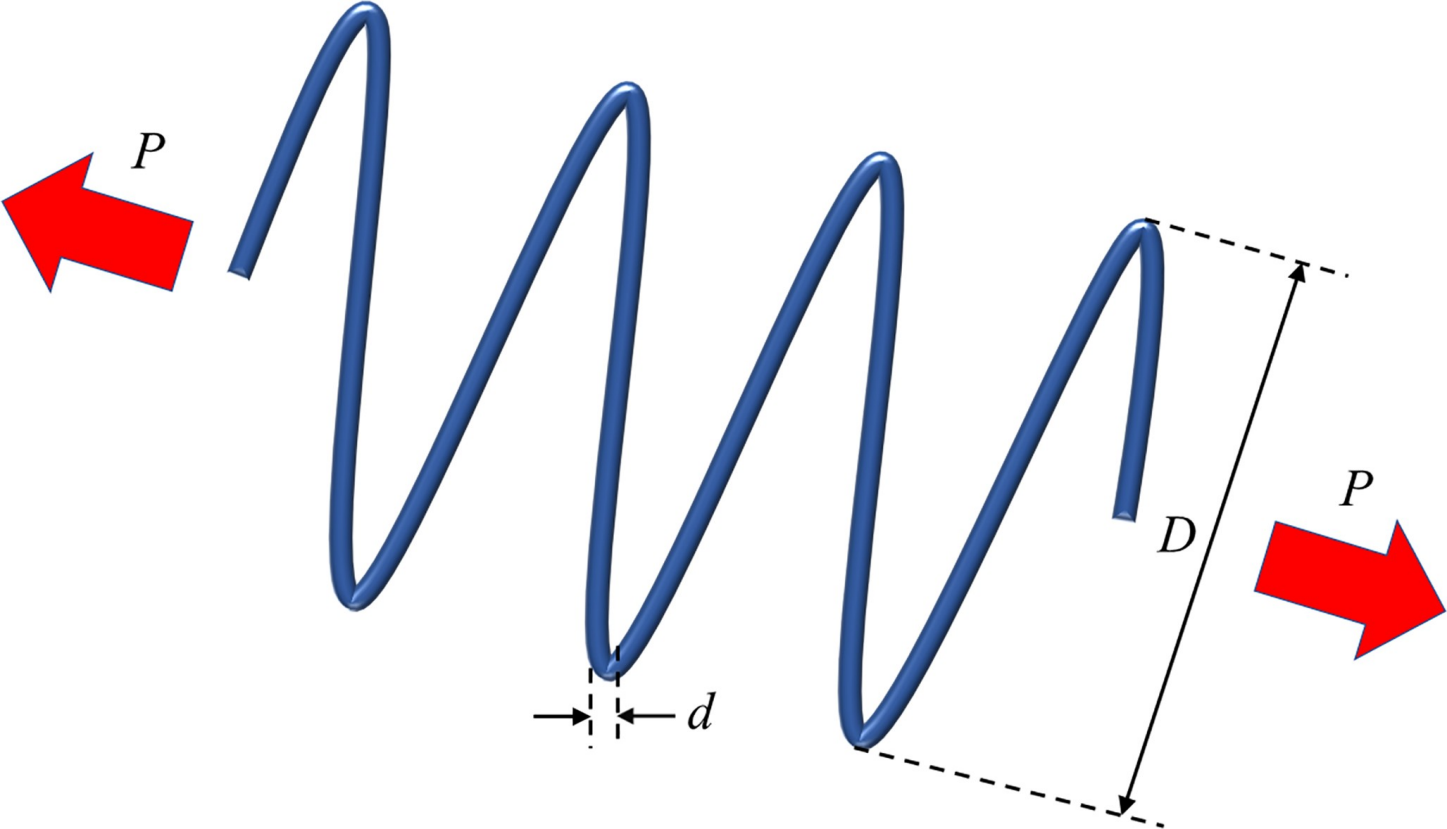
3D schematic view of the compression/tension spring design problem.

As shown in Fig 16, the main goal to be solved in the compression/tension spring design problem is how to select the effective number of active coils (*n*), wire diameter (*d*) and mean coil diameter (*D*) of the spring to minimize the mass of the spring [57].

According to the limitations of physical laws, the compression/tension spring design problem is constrained by shear stress, surge frequency and minimum deflection. If the variables to be optimized are considered as *X* = [*x*_1_, *x*_2_, *x*_3_] = [*d*, *D*, *N*], the problem is described as Eq (29), and its constraints are defined as Eq (30).

$$f(X)=(x_{3}+2)x_{2}x_{1}^{2}$$ (29)

$$\left\{ \begin{array}{c} g_{1}(X)=1-\frac{x_{2}^{3}x_{3}}{71785x_{1}^{4}}\leq 0\\ g_{2}(X)=\frac{4x_{2}^{2}-x_{1}x_{2}}{12566(x_{2}x_{1}^{3}-x_{1}^{4})}+\frac{1}{5108x_{1}^{2}}-1\leq 0\\ g_{3}(X)=1-\frac{140.45x_{1}}{x_{2}^{2}x_{3}}\leq 0\\ g_{4}(X)=x_{1}-x_{4}\leq 0\\ g_{5}(X)=\frac{x_{1}+x_{2}}{1.5}-1\leq 0 \end{array}\right.$$ (30)

were 0.05≤*x*_1_≤2.00, 0.25≤*x*_2_≤1.30, 2.00≤*x*_3_≤15.00.

When TAVOA and the comparison algorithms obtain the best value in the compression/tension spring design problem, the values of each variable are shown in Table 14.

**Table 14:** Statistical results of tension/compression spring problem.

Algorithm	Best	Mean	Worst	SD
GOA	0.012729245	16.67580991	464.8859026	84.7439022
MPA	**0.012665215**	**0.012665244**	**0.012665523**	**5.92448E-08**
PSO	0.012667564	0.013051771	0.015077605	0.000515386
SSA	0.012753639	0.013849355	0.025720296	0.002522712
WOA	0.012665348	0.013600226	0.017774704	0.001208423
AVOA	0.01270936	0.013520448	0.01583301	0.00078102
TAVOA	0.012665538	0.013252806	0.014982031	0.000629048

As seen from Table 14, MPA achieves the best results and is the most stable algorithm in the compression/tension spring design problem. Although TAVOA is not as good as MPA and WOA, it is better than the other five comparison algorithms. In addition, TAVOA is better than AVOA in terms of the best value, the worst value and stability.

Table 15 lists the values of each variable when TAVOA and the comparison algorithms achieve the minimum mass in solving the compression/tension spring design problem.

**Table 15:** Best results of tension/compression spring problem.

Algorithms	Optimum variables			Optimum cost
	*d*	*D*	*N*	
GOA	0.05	0.317269	14.0485	0.012729245
MPA	0.0516876	0.356682	11.291	**0.012665215**
PSO	0.0513313	0.348173	11.8081	0.012667564
SSA	0.053674	0.40638	8.8936	0.012753639
WOA	0.0517716	0.358706	11.1733	0.012665348
AVOA	0.053264	0.39581	9.3179	0.01270936
TAVOA	0.0517784	0.358872	11.1637	0.012665538

**Fig 17:**
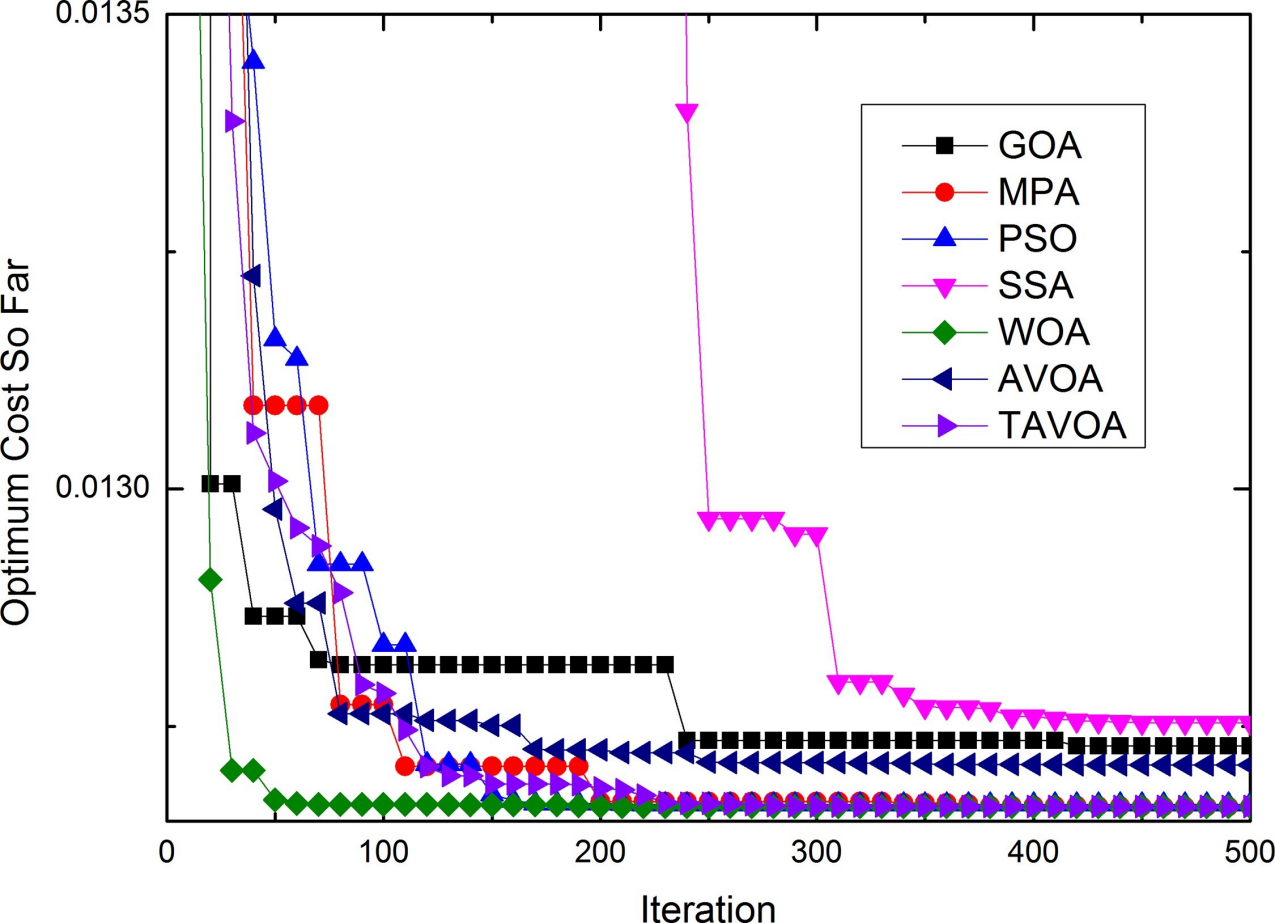
Convergence process in the compression/tension spring design problem.

The convergence curves of TAVOA and comparison algorithms when obtaining the best value in the compression/tension spring design problem are shown in Fig 17.

As seen from Fig 17, the optimum value obtained by TAVOA after 100 iterations is better than AVOA and converges faster than AVOA.

#### 4.4.3 The pressure vessel design problem

**Fig 18:**
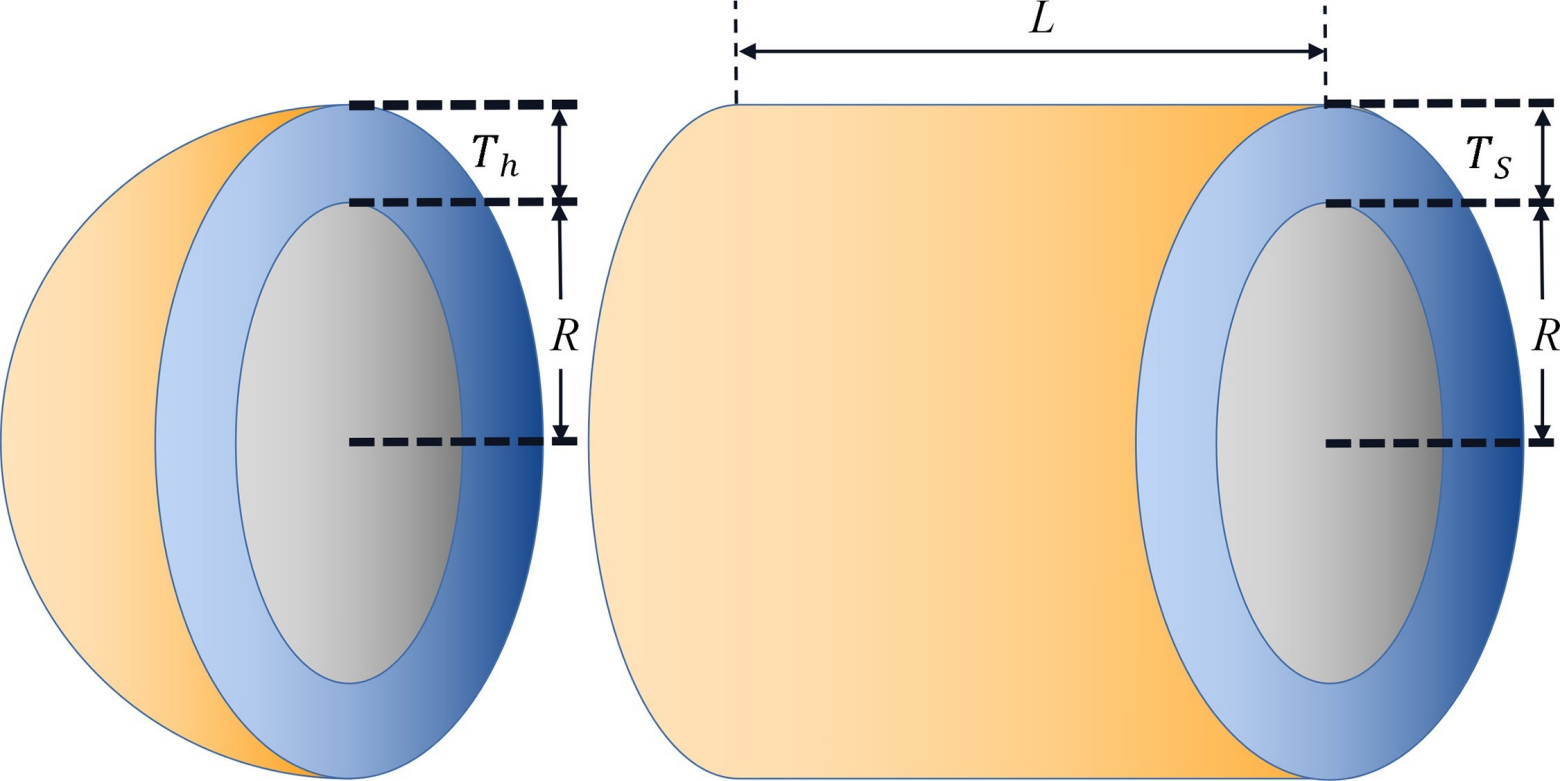
3D schematic view of the pressure vessel design problem.

As shown in Fig 18, the main goal to be solved in the pressure vessel design problem is how to select the thickness of the shell (*T*_*s*_), the thickness of the head (*T*_h_), the radius of the inner circle of the vessel (*R*) and the length of the cylindrical without the head (*L*) to minimize the engineering cost of pressure vessels [58].

According to the limitations of physical laws, the pressure vessel design problem is subject to four linear and nonlinear constraints. If the variables to be optimized are considered as *X* = [*x*_1_, *x*_2_, *x*_3_, *x*_4_] = [*T*_*s*_, *T*_*h*_, *R*, *L*], the problem is described as Eq (31), and its constraints are defined as Eq (32).

$$f(X)=0.6224x_{1}x_{3}x_{4}+1.7781x_{2}x_{3}^{2}+3.1661x_{1}^{2}x_{4}+19.84x_{1}^{2}x_{3}$$ (31)

$$\left\{ \begin{array}{c} g_{1}(X)=-x_{1}+0.0193x_{3}\leq 0\\ g_{2}(X)=-x_{2}+0.00954x_{3}\leq 0\\ g_{3}(X)=-\pi x_{3}^{2}x_{4}-\frac{4}{3}\pi x_{3}^{3}+1296000\leq 0\\ g_{4}(X)=x_{4}-240\leq 0 \end{array}\right.$$ (32)

were 0≤*x*_1_, *x*_2_≤99, 10≤*x*_3_, *x*_4_≤200.

The best value, the worst value, mean value and standard deviation obtained by TAVOA and comparison algorithms in the pressure vessel design problem are shown in Table 16.

**Table 16:** Statistical results of pressure vessel design problem.

Algorithm	Best	Mean	Worst	SD
GOA	4527.338371	5368.644542	6174.355956	428.7217948
MPA	**4527.268027**	**4527.268032**	**4527.268046**	**4.98961E-06**
PSO	4527.27135	6190.273345	7535.014127	1197.423588
SSA	4529.34725	4709.306356	5576.977989	306.5715404
WOA	4907.711687	6338.905597	8248.637632	1025.990865
AVOA	4527.268077	4972.995243	5579.059526	501.086712
TAVOA	**4527.268027**	4905.436585	5579.00791	484.7634125

As seen from Table 16 that TAVOA and MPA achieve the best result in the pressure vessel design problem, but MPA is more stable than TAVOA. In addition, TAVOA is also better than GOA, PSO, WOA and AVOA from the point of view of mean value, the worst value and standard deviation.

Table 17 lists the values of each variable when TAVOA and comparison algorithms achieve the minimum cost in solving the pressure vessel design problem.

**Table 17:** Best results of pressure vessel design problem.

Algorithms	Optimum variables				Optimum cost
	*T*_*s*_	*T*_*h*_	*R*	*L*	
GOA	0.4318173	0.2404585	40.32047	199.9882	4527.338371
MPA	0.4611333	0.2401184	40.31962	200	**4527.268027**
PSO	0.4615843	0.2398836	40.31962	199.9999	4527.27135
SSA	0.4627656	0.2414255	40.34684	199.6214	4529.34725
WOA	0.6598208	0.2810879	44.83852	145.4037	4907.711687
AVOA	0.4611357	0.2400599	40.31962	200	4527.268077
TAVOA	0.4611317	0.2401188	40.31962	200	**4527.268027**

**Fig 19:**
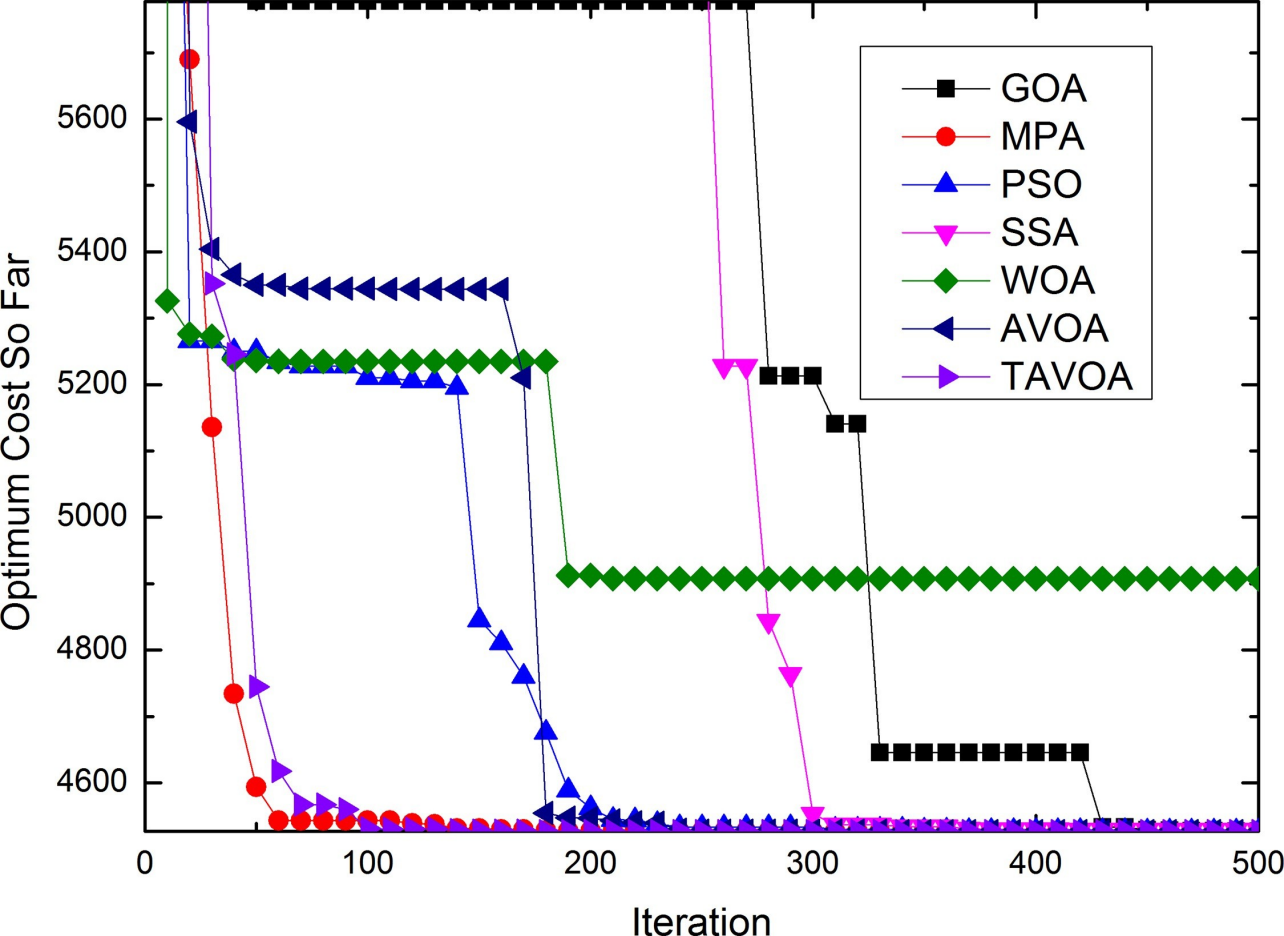
Convergence process in the pressure vessel design problem.

The convergence curves of TAVOA and comparison algorithms when obtaining the best value in the pressure vessel design problem are shown in Fig 19.

As seen from Fig 19, in the pressure vessel design problem, TAVOA performs better than the other six comparison algorithms, and the number of iterations required to reach the best solution is the least.

## 5. Discussion

On the 23 basic benchmark functions, TAVOA performs best on 15 functions compared with AVOA and the other five metaheuristic optimization algorithms. In addition, according to the Wilcoxon rank-sum test, TAVOA is significantly better than AVOA on 13 functions, and the performance of TAVOA is similar to that of AVOA on 5 functions. Especially in unimodal functions and fixed-dimension multi-modal benchmark functions, TAVOA is more prominent.

On the 28 CEC 2013 benchmark functions, TAVOA performs best on 10 functions compared with AVOA and five other metaheuristic optimization algorithms. In addition, according to the Wilcoxon rank-sum test, TAVOA is significantly better than AVOA on 9 functions, and the performance of TAVOA is similar to that of AVOA on 17 functions. Although the performance of TAVOA on the 28 CEC 2013 benchmark functions is not as good as that on the 23 basic benchmark functions, it also greatly improves the performance of AVOA.

Moreover, it can be seen from the convergence curve that TAVOA can obtain better results in many functions at the beginning, which is due to the introduction of the tent chaotic map. When a tent chaotic map is introduced into TAVOA, the population of TAVOA is diversified, and a good feasible solution can be obtained in the early stage. In addition, in the early and middle stages, TAVOA can gradually obtain better feasible solutions because of the introduction of individual history optimal solutions. The introduction of individual history optimal solutions can help individuals to further exploit near their previous positions to find better feasible solutions. At the same time, we can also find that in the later stage, TAVOA can often jump out of the local trap and find a better feasible solution, which is due to the introduction of time-varying mechanism. In the original AVOA, in the exploitation stage, it is considered that the impact of the first group of vultures and the second group of vultures on the current vulture is the same, which makes the exploration ability inferior. Due to the introduction of time-varying mechanism, the exploration ability and exploitation ability of TAVOA can be balanced in such a way that TAVOA still has a certain exploitation ability in the later stage. Therefore, TAVOA can jump out of a locally optimal solution and find a better feasible solution in the later stage.

Moreover, TAVOA still shows good performance in three common real-world engineering problems. TAVOA ranks third in the welded beam design problem and compression/tension spring design problem. In the pressure vessel design problem, TAVOA obtains the same best solution as MPA. It is worth mentioning that in these three real-world engineering problems, TAVOA shows better performance than AVOA. Therefore, it can be concluded that TAVOA also greatly improves the performance of AVOA in real-world engineering problems. TAVOA not only obtains better feasible solutions than AVOA but also converges faster than AVOA.

## 6. Conclusion and future works

In this paper, an improved African vultures optimization algorithm (TAVOA) based on tent chaotic mapping and time-varying mechanism is proposed to improve the African vultures optimization algorithm (AVOA), which makes it possible for TAVOA to be applied to more fields instead of AVOA and obtain better results.

In view of the shortcomings of AVOA, tent chaotic mapping, individual history optimal solution and time-varying mechanism are introduced into TAVOA to enhance the diversity of the TAVOA population, enhance the exploitation ability of TAVOA in the early stage and balance the exploration ability and exploitation ability of TAVOA to enhance the global search ability and local search ability of TAVOA. To verify the effectiveness and efficiency of TAVOA, in addition to AVOA, five state-of-the-art and more studied metaheuristic optimization algorithms are used for comparison. Among 51 benchmark functions, TAVOA performs well in unimodal functions, fixed-dimension multi-modal benchmark functions and composition functions. In addition, experiments in three common real-world engineering problems show that TAVOA greatly improves the performance of AVOA. Besides, the effectiveness of our innovation can also be seen from the experimental convergence curve.

However, TAVOA still has some shortcomings and limitations. First, the selection of tent chaotic map is based on published papers and experience. The most suitable chaotic map is not selected by applying all the commonly used chaotic maps to AVOA. Second, in the time-varying mechanism, the parameters are obtained according to experience, and the weight factors are also designed according to experience. Therefore, the influence of the parameters on the performance of the algorithm is not considered. Third, although TAVOA performs particularly well on 23 commonly used benchmark functions, the performance of TAVOA is slightly insufficient on the multi-modal function of the CEC 2013 benchmark function. Therefore, in future work, the commonly used chaotic maps can be applied to TAVOA, and the most appropriate chaotic map can be selected in different application scenarios. In addition, the influence of the parameters in the time-varying mechanism of the algorithm can be considered, and the adaptive weight factors can be designed. Finally, more targeted strategies can be designed to improve the performance of TAVOA in multi-modal functions.

## Supporting information

S1 Dataset Benchmark functions used in this paper. (DOCX)
