# Supplementary material for: An improved African vultures optimization algorithm based on tent chaotic mapping and time-varying mechanism
Source: PLoS One. 2021 Nov 30;16(11):e0260725. doi: 10.1371/journal.pone.0260725 (PMC8631685; doi:10.1371/journal.pone.0260725)
Supplement: S1 Dataset — (DOCX) [file pone.0260725.s001.docx]

**S1 Dataset. Benchmark Functions used in this paper**

**Table 1. Details of 23 basic benchmark functions.**

| Type | Function | Dim | Range | $\boldsymbol{f}_{\boldsymbol{min}}$ |
| --- | --- | --- | --- | --- |
| Unimodal benchmark function | $f1\left( x \right)=\sum_{i=1}^{Dim} x_{i}^{2}$ | 30 | $\left[ -100,100 \right]^{Dim}$ | 0 |
|  | $f2\left( x \right)=\sum_{i=1}^{Dim} \left\vert x_{i} \right\vert+\prod_{i=1}^{Dim} \left\vert x_{i} \right\vert$ | 30 | $\left[ -10,10 \right]^{Dim}$ | 0 |
|  | $f3\left( x \right)=\sum_{i=1}^{Dim} \left( \sum_{j=1}^{i} x_{j} \right)^{2}$ | 30 | $\left[ -100,100 \right]^{Dim}$ | 0 |
|  | $f4\left( x \right)={max}_{i}\left\{ \left\vert x_{i} \right\vert,1\leq i\leq Dim \right\}$ | 30 | $\left[ -100,100 \right]^{Dim}$ | 0 |
|  | $f5\left( x \right)=\sum_{i=1}^{Dim-1} \left[ {100\left( x_{i+1}-x_{i}^{2} \right)}^{2}+\left( x_{i}-1 \right)^{2} \right]$ | 30 | $\left[ -30,30 \right]^{Dim}$ | 0 |
|  | $f6\left( x \right)=\sum_{i=1}^{Dim} \left( x_{i}+0.5 \right)^{2}$ | 30 | $\left[ -100,100 \right]^{Dim}$ | 0 |
|  | $f7\left( x \right)=\sum_{i=1}^{Dim} ix_{i}^{4}+random[0,1)$ | 30 | $\left[ -1.28,1.28 \right]^{Dim}$ | 0 |
| Multi-modal benchmark function | $f8\left( x \right)=\sum_{i=1}^{Dim} -x_{i}sin\left( \sqrt{\left\vert x_{i} \right\vert} \right)$ | 30 | $\left[ -500,500 \right]^{Dim}$ | $-418.9829\times Dim$ |
|  | $f9\left( x \right)=\sum_{i=1}^{Dim} \left[ x_{i}^{2}-10cos\left( 2\pi x_{i} \right)+10 \right]$ | 30 | $\left[ -5.12,5.12 \right]^{Dim}$ | 0 |
|  | $f10(x)=-20exp\left( -0.2\sqrt{\frac{\sum_{i=1}^{Dim} x_{i}^{2}}{Dim}} \right)-exp\left( \frac{\sum_{i=1}^{Dim} cos\left( 2\pi x_{i} \right)}{Dim} \right)+20+e$ | 30 | $\left[ -32,32 \right]^{Dim}$ | 0 |
|  | $f11\left( x \right)=\frac{1}{4000}\sum_{i=1}^{Dim} x_{i}^{2}-\prod_{i=1}^{Dim} cos\left( \frac{x_{i}}{\sqrt{i}} \right)+1$ | 30 | $\left[ -600,600 \right]^{Dim}$ | 0 |
|  | $f12\left( x \right)=\left\{ 10sin\left( \pi y_{1} \right)+\sum_{i=1}^{Dim-1} \left( y_{i}-1 \right)^{2}\left[ 1+10{sin}^{2}\left( \pi y_{i+1} \right) \right]-\left( y_{Dim}-1 \right)^{2} \right\}+\sum_{i=1}^{Dim} u\left( x_{i},5,100,4 \right)$  $y_{i}=1+(x_{i}+1)/4 u\left( x_{i},a,k,m \right)=\left\{ \begin{aligned} k\left( x_{i}-a \right)^{m}, x_{i}>a \\ 0, -a<x_{i}<a \\ {k\left( -x_{i}-a \right)}^{m}, x_{i}<-a \end{aligned} \right.$ | 30 | $\left[ -50,50 \right]^{Dim}$ | 0 |
|  | $f13\left( x \right)=0.1\left\{ {sin}^{2}\left( 3\pi x_{i} \right)+\sum_{i=1}^{Dim} \left( x_{i}-1 \right)^{2}\left[ 1+{sin}^{2}\left( 3\pi x_{i}+1 \right) \right]+\left( x_{Dim}-1 \right)^{2}\left[ 1+{sin}^{2}\left( 2\pi x_{Dim} \right) \right] \right\}+\sum_{i=1}^{Dim} u\left( x_{i},5,100,4 \right)$ | 30 | $\left[ -50,50 \right]^{Dim}$ | 0 |
| Fixed-dimension multi-modal benchmark function | $f14\left( x \right)=\left[ \frac{1}{500}+\sum_{j=1}^{25} \frac{1}{j+\sum_{i=1}^{2} \left( x_{i}-a_{ij} \right)^{6}} \right]^{-1}$ | 2 | $\left[ -65,65 \right]^{Dim}$ | 1 |
|  | $f15\left( x \right)=\sum_{i=1}^{11} \left[ a_{i}-\left[ x_{1}\left( b_{i}^{2}+b_{i}x_{2} \right) \right]/\left( b_{i}^{2}+b_{i}x_{3}+x_{4} \right) \right]^{2}$ | 4 | $\left[ -5,5 \right]^{Dim}$ | 0.00030 |
|  | $f16\left( x \right)=4x_{1}^{2}-2.1x_{1}^{4}+1/3x_{1}^{6}+x_{1}x_{2}-4x_{2}^{2}+4x_{2}^{4}$ | 2 | $\left[ -5,5 \right]^{Dim}$ | -1.0316 |
|  | $f17\left( x \right)=\left( x_{2}-{5.1x_{1}^{2}}/{4\pi^{2}}+{5x_{1}}/\pi-6 \right)^{2}+10\left( 1-1/{8\pi} \right)cosx_{1}+10$ | 2 | $\left[ -5,5 \right]^{Dim}$ | 0.398 |
|  | $f18\left( x \right)=\left[ 1+\left( x_{1}+x_{2}+1 \right)^{2}\left( 19-14x_{1}+3x_{1}^{2}-14x_{2}+6x_{1}x_{2}+3x_{2}^{2} \right) \right]\times\left[ 30+\left( 2x_{1}-3x_{2} \right)^{2}\left( 18-32x_{1}+12x_{1}^{2}+48x_{2}-36x_{1}x_{2}+27x_{2}^{2} \right) \right]$ | 2 | $\left[ -2,2 \right]^{Dim}$ | 3 |
|  | $f19\left( x \right)=-\sum_{i=1}^{4} c_{i}exp(-\sum_{j=1}^{3} {a_{ij}\left( x_{i}-p_{ij} \right)}^{2})$ | 3 | $\left[ 1,3 \right]^{Dim}$ | -3.86 |
|  | $f20\left( x \right)=-\sum_{i=1}^{4} c_{i}exp(-\sum_{j=1}^{6} {a_{ij}\left( x_{i}-p_{ij} \right)}^{2})$ | 6 | $\left[ 0,1 \right]^{Dim}$ | -3.32 |
|  | $f21\left( x \right)=-\sum_{i=1}^{5} \left[ \left( X-a_{i} \right)\left( X-a_{i} \right)^{T}+c_{i} \right]^{-1}$ | 4 | $\left[ 0,10 \right]^{Dim}$ | -10.1532 |
|  | $f22\left( x \right)=-\sum_{i=1}^{7} \left[ \left( X-a_{i} \right)\left( X-a_{i} \right)^{T}+c_{i} \right]^{-1}$ | 4 | $\left[ 0,10 \right]^{Dim}$ | -10.4028 |
|  | $f23\left( x \right)=-\sum_{i=1}^{10} \left[ \left( X-a_{i} \right)\left( X-a_{i} \right)^{T}+c_{i} \right]^{-1}$ | 4 | $\left[ 0,10 \right]^{Dim}$ | -10.5363 |

**Table 2. Details of 28 CEC 2013 benchmark functions.**

| Type | Function | Name | Dim | Range | $\boldsymbol{f}_{\boldsymbol{min}}$ |
| --- | --- | --- | --- | --- | --- |
| Unimodal benchmark function | $f24$ | Sphere Function | 30 | $\left[ -100,100 \right]^{Dim}$ | -1400 |
|  | $f25$ | Rotated High Conditioned Elliptic Function | 30 | $\left[ -100,100 \right]^{Dim}$ | -1300 |
|  | $f26$ | Rotated Bent Cigar Function | 30 | $\left[ -100,100 \right]^{Dim}$ | -1200 |
|  | $f27$ | Rotated Discus Function | 30 | $\left[ -100,100 \right]^{Dim}$ | -1100 |
|  | $f28$ | Different Powers Function | 30 | $\left[ -100,100 \right]^{Dim}$ | -1000 |
| Multi-modal benchmark function | $f29$ | Rotated Rosenbrock’s Function | 30 | $\left[ -100,100 \right]^{Dim}$ | -900 |
|  | $f30$ | Rotated Schaffers F7 Function | 30 | $\left[ -100,100 \right]^{Dim}$ | -800 |
|  | $f31$ | Rotated Ackley’s Function | 30 | $\left[ -100,100 \right]^{Dim}$ | -700 |
|  | $f32$ | Rotated Weierstrass Function | 30 | $\left[ -100,100 \right]^{Dim}$ | -600 |
|  | $f33$ | Rotated Griewank’s Function | 30 | $\left[ -100,100 \right]^{Dim}$ | -500 |
|  | $f34$ | Rastrigin’s Function | 30 | $\left[ -100,100 \right]^{Dim}$ | -400 |
|  | $f35$ | Rotated Rastrigin’s Function | 30 | $\left[ -100,100 \right]^{Dim}$ | -300 |
|  | $f36$ | Non-Continuous Rotated Rastrigin’s Function | 30 | $\left[ -100,100 \right]^{Dim}$ | -200 |
|  | $f37$ | Schwefel's Function | 30 | $\left[ -100,100 \right]^{Dim}$ | -100 |
|  | $f38$ | Rotated Schwefel's Function | 30 | $\left[ -100,100 \right]^{Dim}$ | 100 |
|  | $f39$ | Rotated Katsuura Function | 30 | $\left[ -100,100 \right]^{Dim}$ | 200 |
|  | $f40$ | Lunacek Bi_Rastrigin Function | 30 | $\left[ -100,100 \right]^{Dim}$ | 300 |
|  | $f41$ | Rotated Lunacek Bi_Rastrigin Function | 30 | $\left[ -100,100 \right]^{Dim}$ | 400 |
|  | $f42$ | Expanded Griewank’s plus Rosenbrock’s Function | 30 | $\left[ -100,100 \right]^{Dim}$ | 500 |
|  | $f43$ | Expanded Scaffer’s F6 Function | 30 | $\left[ -100,100 \right]^{Dim}$ | 600 |
| composition benchmark function | $f44$ | Composition Function 1 (n=5,Rotated) | 30 | $\left[ -100,100 \right]^{Dim}$ | 700 |
|  | $f45$ | Composition Function 2 (n=3,Unrotated) | 30 | $\left[ -100,100 \right]^{Dim}$ | 800 |
|  | $f46$ | Composition Function 3 (n=3,Rotated) | 30 | $\left[ -100,100 \right]^{Dim}$ | 900 |
|  | $f47$ | Composition Function 4 (n=3,Rotated) | 30 | $\left[ -100,100 \right]^{Dim}$ | 1000 |
|  | $f48$ | Composition Function 5 (n=3,Rotated) | 30 | $\left[ -100,100 \right]^{Dim}$ | 1100 |
|  | $f49$ | Composition Function 6 (n=5,Rotated) | 30 | $\left[ -100,100 \right]^{Dim}$ | 1200 |
|  | $f50$ | Composition Function 7 (n=5,Rotated) | 30 | $\left[ -100,100 \right]^{Dim}$ | 1300 |
|  | $f51$ | Composition Function 8 (n=5,Rotated) | 30 | $\left[ -100,100 \right]^{Dim}$ | 1400 |
